# SRT-Server: powering the analysis of spatial transcriptomic data

**DOI:** 10.1186/s13073-024-01288-6

**Published:** 2024-01-26

**Authors:** Sheng Yang, Xiang Zhou

**Affiliations:** 1 Department of Biostatistics, Center for Global Health, School of Public Health, Nanjing Medical University, Nanjing, Jiangsu 211166 China; 2 Department of Biostatistics, School of Public Health, University of Michigan, Ann Arbor, MI 48109 USA; 3 Center for Statistical Genetics, University of Michigan, Ann Arbor, MI 48109 USA

**Keywords:** Spatial resolved transcriptomics (SRT), Data analysis, Visualization, Analysis flow chart, Web server

## Abstract

**Background:**

Spatial resolved transcriptomics (SRT) encompasses a rapidly developing set of technologies that enable the measurement of gene expression in tissue while retaining spatial localization information. SRT technologies and the enabled SRT studies have provided unprecedent insights into the structural and functional underpinnings of complex tissues. As SRT technologies have advanced and an increasing number of SRT studies have emerged, numerous sophisticated statistical and computational methods have been developed to facilitate the analysis and interpretation of SRT data. However, despite the growing popularity of SRT studies and the widespread availability of SRT analysis methods, analysis of large-scale and complex SRT datasets remains challenging and not easily accessible to researchers with limited statistical and computational backgrounds.

**Results:**

Here, we present SRT-Server, the first webserver designed to carry out comprehensive SRT analyses for a wide variety of SRT technologies while requiring minimal prior computational knowledge. Implemented with cutting-edge web development technologies, SRT-Server is user-friendly and features multiple analytic modules that can perform a range of SRT analyses. With a flowchart-style interface, these different analytic modules on the SRT-Server can be dragged into the main panel and connected to each other to create custom analytic pipelines. SRT-Server then automatically executes the desired analyses, generates corresponding figures, and outputs results—all without requiring prior programming knowledge. We demonstrate the advantages of SRT-Server through three case studies utilizing SRT data collected from two common platforms, highlighting its versatility and values to researchers with varying analytic expertise.

**Conclusions:**

Overall, SRT-Server presents a user-friendly, efficient, effective, secure, and expandable solution for SRT data analysis, opening new doors for researchers in the field. SRT-Server is freely available at https://spatialtranscriptomicsanalysis.com/.

**Supplementary Information:**

The online version contains supplementary material available at 10.1186/s13073-024-01288-6.

## Background

Spatially resolved transcriptomics (SRT) encompasses a suite of innovative technologies that enable gene expression profiling of complex tissues with spatial localization information [1–4]. Some of these technologies, such as 10 × Visium [5], Slide-seqV2 [6], and Stereo-seq [7], are based on high-throughput sequencing, while others like MERFISH [8], seqFISH [9], and CosMx [10] rely on single-molecule fluorescent in situ hybridization. As these technologies advance rapidly, numerous computational methods and software tools have been developed to facilitate various analyses in SRT studies [11–18]. These tools are designed for tasks such as detecting spatially variable genes (SVGs) [19–21], conducting cell typing and cell type clustering for single-cell resolution SRT studies [22–25], performing cell type deconvolution for spot resolution SRT [26], characterizing spatial domains within tissues [22, 27], inferring cell–cell communication [13, 28, 29], identifying differentially expressed genes (DEGs) in specific cell types or spatial domains [30, 31], carrying out pathway enrichment analysis [32], and reconstructing pseudo-time trajectories across cells or spatial locations [33]. Collectively, these SRT experimental and computational technologies have revolutionized numerous fields of biology, allowing for comprehensive characterization of the transcriptomic and functional landscape of complex tissues, and providing new biological insights.

Despite the growing popularity of SRT studies and the widespread availability of SRT analysis software, analysis of large-scale and complex SRT datasets remains challenging and not easily accessible to researchers with limited statistical and computational backgrounds. Specifically, SRT computational methods and software tools can often be difficult to use, requiring considerable statistical knowledge and computational skills for effective application. Challenges include choosing the proper methods for analysis, installing new software, building the corresponding computational environment, resolving package dependency conflicts, determining the appropriate command line for the intended analysis, and debugging code whenever error arises. This task can be daunting even for experienced bioinformaticians, especially when faced with administrative restrictions on the local computing cluster.

To make SRT data analysis fully accessible to mainstream biologists, we have developed SRT-Server, the first webserver designed to carry out a comprehensive set of SRT analyses for a wide variety of SRT technologies, while requiring minimal prior computational knowledge. SRT-Server is user-friendly, features ten analytic modules that can perform all the SRT analyses mentioned earlier in the introduction, and differs substantially from existing analytic platforms (Table 1, Additional file 1: Table S1 and S2; more details in the Discussion and Additional file 2). We demonstrate the benefits of SRT-Server through the analysis of three example SRT datasets collected from different technologies, tissues, and species. Overall, we believe SRT-Server will prove invaluable to the SRT research community, facilitating effective, and comprehensive analysis of the ever-growing collection of SRT datasets.

**Table 1:** Summary of the analytic tools for SRT data analysis^a^

	**SRT-Server**	**Giotto**	**Squidpy**	**Seurat**	**SPATA2**	**10 × Platform**	**spatialGE**	**Spaniel**	**STellaris**
**Implemented language**	R + python	R	python	R	R		R	R	R + python
**Generic SRT format**	Yes	Yes	Yes	Yes	Yes	No	Yes	Yes	Yes
**Specific SRT format**	Yes	Yes	Yes	Yes	Yes	Yes	No	No	No
**Interactive operation**	Yes	No	No	No	Yes	Yes	No	No	No
**Platform specificity**	Yes	No	No	No	No	Yes	No	No	No
**Visualization**	Yes	Yes	Yes	Yes	Yes	Yes	Yes	Yes	Yes
**Analytic modules**	Yes								
QC	Yes	Yes	Yes	Yes	Yes	Yes	Yes	Yes	Yes
SVG	Yes	Yes	Yes	No	Yes	Yes	No	No	No
DECON	Yes	Yes	No	No	Yes	Yes	Yes	No	Yes
Clustering	Yes	Yes	Yes	Yes	Yes	Yes	Yes	Yes	No
CCC	Yes	Yes	Yes	No	No	No	No	No	No
DE	Yes	Yes	No	Yes	Yes	Yes	No	No	No
TRAJ	Yes	No	No	No	Yes	No	No	No	No
ORA	Yes	No	No	No	No	No	No	No	No
**Reference**	N/A	[13]	[11]	[30]	[14]	N/A	[15]	[16]	[17]

^a^: All URLs for the existing methods: Giotto: https://rubd.github.io/Giotto_site/; Squidpy: https://squidpy.readthedocs.io/en/latest/; Seurat: https://satijalab.org/seurat/; SPATA2: https://themilolab.github.io/SPATA2/; 10 × Platform: https://support.10xgenomics.com/spatial-gene-expression/software/pipelines/latest/algorithms/overview; spatialGE: https://github.com/FridleyLab/spatialGE; Spaniel: https://github.com/RachelQueen1/Spaniel; STellaris: https://spatial.rhesusbase.com/

## Implementation

### Design of SRT-Server

**Fig. 1:**
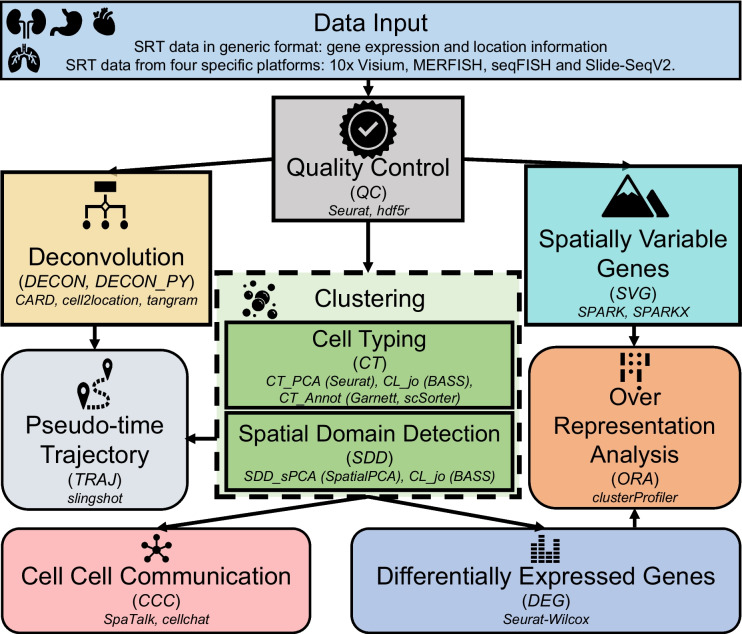
Ten analytic modules and their hierarchical relationship in SRT-Server. SRT-Server features ten interconnected analytic modules (boxes), each containing one or more analytic methods (small font letters). Users can connect these modules in a hierarchical manner to form a customized analytic pipeline. The pipeline always begins with user-uploaded data in a generic or platform-specific format. Next, the QC module performs quality control and saves the processed data in h5 format. From the QC module, the pipeline branches into five analytic modules: the DECON module, which uses CARD to estimate cell type compositions on each measured spot through deconvolution; the DECON_PY module, which uses cell2location and tangram to perform deconvolution; the SVG module, which identifies spatial variable genes (SVGs) with SPARK or SPARK-X; the CT module, which clusters cells for single-cell resolution SRT using Seurat or BASS, or annotates them using scSorter or Garnett; and the SDD module, which clusters spatial domains using SpatialPCA or BASS. From the clustering modules (CT or SDD), the pipeline moves to three additional analytic modules: the TRAJ module for pseudo-time inference of cell types or spatial domains; the CCC module for detecting cell–cell or domain-domain interactions using a ligand-receptor database provided on the SRT-Server; and the DEG module for identifying differentially expressed genes (DEGs) based on the detected cell types or spatial domains. For genes output from the SVG or DEG modules, the ORA module estimates enrichment of biological pathways using five pathway databases provided on the SRT-Server. Note that regular rectangular boxes represent upstream analysis (procedures before CT), while rounded rectangular boxes represent downstream analysis based on clustering results

SRT-Server (https://spatialtranscriptomicsanalysis.com/) is a cutting-edge webserver that utilizes state-of-the-art web technologies and analytical methods to provide comprehensive and user-friendly analysis for SRT datasets. It allows users to input their SRT data in either a generic SRT data format or a platform-specific SRT data format obtained from commonly used SRT platforms (Fig. 1). The generic SRT data format comprises two files: a gene expression file containing gene expression measurements across spatial locations measured on the tissue, and a location file containing the *x* and *y* coordinates of the measured locations. Platform-specific data formats are available in the form of raw data produced by four commonly used SRT platforms, including 10 × Visium [5], VIZGEN MERFISH [8], seqFISH [34], and Slide-SeqV2 [6]. In addition to SRT data, single-cell RNA sequencing (scRNA-seq) data may be necessary for certain analytic tasks such as reference-based cell type deconvolution. The input for scRNA-seq data includes an expression count matrix that contains gene expression measurements across single cells and a meta matrix containing cell type annotations for each cell.

SRT-Server provides a user-friendly interface, allowing users to design their desirable analytic pipelines on the input data by selecting and combining distinct SRT analytic modules. Currently, SRT-Server comprises ten SRT analytic modules, each of which can perform a specific analytic task such as quality control, detecting SVGs, cell typing/cell type clustering in single-cell resolution SRT, cell type deconvolution in spot resolution SRT, detecting spatial domains, identifying genes that are DEGs in a specific cell type or spatial domain, pathway enrichment analysis with the identified DEGs or SVGs, detecting cell–cell communications in both single-cell resolution and spot resolution SRT, and estimating pseudo-time trajectories across cells or spatial locations. Each analytic module in SRT-Server contains multiple SRT computational methods previously developed for the analytic task, providing users with the flexibility to select the best computational method to suit their needs.

To ensure code compatibility and transportability, each analytic module in SRT-Server follows the same coding standard and consists of three main functions: *check*, *call*, and *post*. These functions are called sequentially by the application programming interfaces (APIs). In the *check* function, SRT-Server verifies the inputs for the module, saves all specified model parameters and transfers to the data format for *call* function. The *call* function carries out analysis by fitting the model, saves temporary files to the disk, and reports the path of the output results. In the *post* function, SRT-Server loads the temporary files and processes them to make them available to the user. The three main functions are carried out in a sequential fashion to minimize computational resource requirements. The three main functions in different modules are also relatively independent of each other, allowing for efficient concurrent computation. In addition to the three main functions, some analytic modules may include a *plot* function that generates figures and saves R plotting objects. The details of each analytic module are described in the module section below.

The analytic modules in SRT-Server provide the foundation for building user-defined analytic pipelines. These modules are represented as icons on the sidebar in the graphical interface of the SRT-Server. Users can drag these modules onto a canvas on the interface and use arrows to connect them to form their desired analytic pipeline. Therefore, the entire process of building the pipeline is user-friendly and interactive. In addition, users can select a particular method inside each module for analysis. For each selected method, users can choose to use pre-specified default parameters in the method or specify the modeling parameters as desired. With the user-defined pipeline, SRT-Server automatically carries out the desired analyses, generates corresponding figures, and outputs results for users to download. In addition, SRT-Server builds upon a high-performance concurrent programming framework, allowing each analytic task to be assigned to a different node and interconnected with each other using disk path. Such coding framework ensures efficient and effective computation.

Overall, the module framework and user-friendly interface of SRT-Server allows users to build analytic pipelines in an intuitive manner and carry out comprehensive analyses of SRT data without prior programming skills. Furthermore, the framework of SRT-Server is expandable, allowing for easy incorporation of future software tools as new modules for SRT analysis. To incorporate a new module into SRT-Server, developers only need to provide the code for the module in SRT-Server standard code formatting, along with any additional information for the dependency structure of the software and package. The developers can send email inquiry or create an issue on GitHub page to initiate the new method or new module implementation process. Upon verifying the code of the new method/module, it will be implemented in SRT-Server in a timely manner. With this expandable framework, SRT-Server can incorporate any future software tools to enhance the capabilities of its analytic modules, providing users with even more options for comprehensive analytics.

### Technical implementation of SRT-Server

**Fig. 2:**
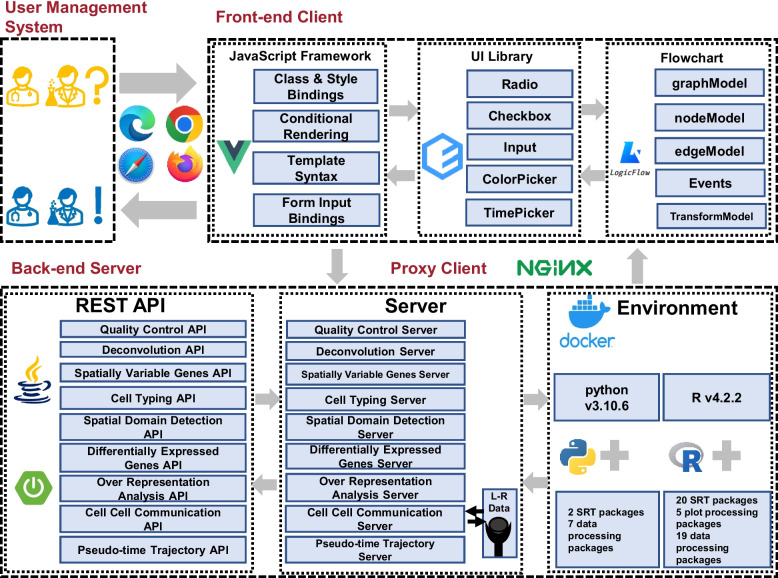
The design and implementation of SRT-Server. SRT-Server is a web-based application with an easy-to-use interface designed for analyzing and visualizing spatial transcriptomics data. Users can access SRT-Server website using Chrome or Firefox browsers. After logging in, users can upload their spatial transcriptomics data, create a personalized analytic pipeline, carry out desired SRT analyses, and download the generated results and figures. SRT-Server consists of a user management system, a front-end client, a back-end server, and a proxy client. The front-end client utilizes the VUE JavaScript framework, Element UI library, and LogicFlow for analytic pipeline creation. The back-end server employs the Spring Boot framework to build the ten analytic modules mentioned in the previous figure. The server further uses Docker to create an environment that encapsulates the R computing environment (v4.2.2) along with 43 R packages and the python computing environment (v3.10.6) along with 7 R packages. We used APIs to transfer ten servers. Overall, SRT-Server facilitates effective and comprehensive analysis of the ever-growing collection of SRT datasets

SRT-Server is designed as a client–server website to provide a secure, efficient, stable, and user-friendly server environment (Fig. 2). It has been thoroughly tested on multiple web browsers including Chrome, Firefox, Microsoft Edge, and Safari, as well as on three distinct operating systems including Windows, MacOS, and Linux. SRT-Server comprises four major technical components: the user management system, the frontend web application, the backend web server, and the proxy client. These components work together seamlessly to provide a user-friendly and computationally efficient experience for users analyzing SRT data.

The user management system in SRT-Server is responsible for managing user registration and login, recording user-built analysis pipelines, and outputting results from different projects. It employs three web frameworks: Spring Security, Druid, and MySQL. Spring Security is a robust and customizable authentication and access-control framework that SRT-Server uses to provide comprehensive support for both authentication and authorization. It ensures the security of user data and restricts access to unauthorized users. Druid is a high-performance real-time analytics database that SRT-Server uses to reduce the time to load, manage, and query data while supporting high query concurrency. It provides fast and reliable data access, making SRT-Server’s data processing and retrieval efficient and effective. MySQL is a powerful and stable solution used in SRT-Server for ease of data management. It is a widely used, open-source relational database management system that ensures the security and reliability of data storage and retrieval. Together, these web frameworks provide a stable and secure foundation for SRT-Server’s user management system, ensuring the confidentiality and integrity of user data and making the platform a reliable and efficient tool for SRT data analysis.

The frontend web application in SRT-Server is responsible for data uploading, interactive analytic pipeline building, model parameter setup, and results reporting and downloading. It is designed as a web single page application (SPA) and implemented using three open-source frameworks: VUE, Element-UI, and LogicFlow. VUE is a rich and progressive ecosystem that builds on top of standard HTML, CSS, and JavaScript with an intuitive API that underlies the framework of SRT-Server. It provides a flexible and easy-to-use platform for building user-friendly interfaces that streamline data analysis. Element-UI is a comprehensive UI library that provides different choices of parameters for different analysis procedures in SRT-Server. It offers a wide range of user interface components such as radio buttons, checkboxes, input fields, time pickers, color pickers, and cascader, making it easy for users to set up their desired model parameters, select color scheme in the plots, and select files for uploading. LogicFlow is a flexible flow chart framework that provides a Lego-like interface for building analytic pipelines in SRT-Server. It enables users to drag and drop analytic modules and connect them together to form a user-defined analytic pipeline. This framework simplifies the process of building an analytic pipeline, making it more accessible to users without prior programming experience. Together, these open-source frameworks provide a solid foundation for SRT-Server’s frontend web application, allowing for easy and efficient data analysis while providing a user-friendly interface.

The web frontend in SRT-Server communicates with the Java-powered backend server through asynchronous HTTP requests (AJAX) using JSON as an interchangeable format. All transmissions between the frontend and backend are encrypted using a secure socket layer (SSL) to ensure the confidentiality and integrity of user data. The Nginx proxy acts as a mediator, accepting HTTP requests from the website and forwarding them to the web frontend graphical user interface (GUI) and/or backend application based on URL routing rules. This setup ensures efficient and reliable communication between the frontend and backend of SRT-Server. The client–server communication in SRT-Server is based on RESTful APIs constructed using Spring Boot. There are eight analysis procedure APIs available, providing a flexible and customizable platform for users to analyze their SRT data. Together, these technologies provide a robust and secure framework for SRT-Server, ensuring efficient and effective communication between the frontend and backend while maintaining the confidentiality and integrity of user data.

The backend web server in SRT-Server is responsible for detailed computation and is based on three open-source frameworks: Nacos, Sentinel, and PowerJob. Nacos supports a dynamic configuration service, allowing the server administrator of SRT-Server to manage the configuration of all applications and services in a centralized, externalized, and dynamic manner across different environments. This framework ensures the smooth operation of SRT-Server and allows for easy management and scalability of the server. Sentinel uses flow control, flow shaping, circuit breaking, and system adaptive protection to ensure the stability and robustness of SRT-Server’s microservices. This framework helps to prevent system overload and ensures the efficient and effective operation of SRT-Server. SRT-Server uses PowerJob, a distributed computing and job scheduling framework, to perform debugging for different analytic modules one at a time. This framework ensures efficient computation and minimizes the risk of errors in the analysis process. The backend server deploys Docker to resolve conflicts between software packages. Docker provides an R (v4.2.2) and python (v.3.10.6) along with 53 third-party packages for the ten analytic modules and eight visualization modules. These packages include 26 data processing packages, five plot processing packages, and 22 SRT analysis packages (Table 2). For example, the SVG module contains four third-party SRT packages including SPARK [19, 20], amap, dplyr, and spdep. These packages ensure efficient and accurate computation for SRT analysis, making SRT-Server a reliable and powerful tool for data analysis. Together, these frameworks and technologies provide a robust and efficient backend for SRT-Server, ensuring the smooth operation of the server and the accurate and reliable analysis of SRT data.

**Table 2:** Included R and python packages for different modules in SRT-Server

Modules/Utilities	Packages
Input/Output (IO)	hdf5r
Quality Control (QC)	Seurat, dplyr, bigreadr
Deconvolution (DECON)	CARD, hdf5r, plyr, gtools bigreadr, dplyr, SingleCellExperiment
Deconvolution (DECON_py)	os, numpy, pandas, anndata, h5py, scipy, re, cell2location, tangram
DECON_Plot	hdf5r, dplyr, bigreadr, ggplot2, scatterpie, SingleCellExperiment
Spatially Variable Genes (SVG)	SPARK, dplyr, amap, spdep
SVG_Plot	Seurat, ggplot2, dplyr, viridis, tidyr, reshape2, scales
Cell Typing (CT)	Seurat, SeuratDisk, BASS, scSorter, monocle, garnett, org.Hs.eg.db, org.Mm.eg.db, glmGamPoi, dplyr, hdf5r, plyr
Spatial Domain Detection (SDD)	BASS, SpatialPCA, dplyr
CT_Plot and SDD_Plot	Seurat, hdf5r, dplyr, bigreadr, ggplot2
Differentially Expressed Genes (DE)	Seurat, hdf5r, dplyr, bigreadr
DE_Plot	hdf5r, dplyr, bigreadr ComplexHeatmap, viridis, circlize, reshape2
Cell Cell Communication (CCC)	hdf5r, SpaTalk, liana, bigreadr, dplyr, SingleCellExperiment, Giotto
CCC_Plot	CellChat, bigreadr, dplyr
Over Representative Analysis (ORA)	clusterProfiler, ReactomePA, DOSE, org.Mm.eg.db, org.Hs.eg.db, dplyr, tibble, Matrix, bigreadr
Psudo-time Trajectory (TRAJ)	Slingshot, SingleCellExperiment, tradeSeq, BiocParallel, Seurat, hdf5r, stringr, dplyr, tibble, tidyr, bigreadr
TRAJ_Plot	hdf5r, ggplot2, bigreadr, dplyr, ComplexHeatmap, viridis, circlize, tidyr, reshape2, scales, ggtree, aplot, patchwork

Finally, the proxy client is deployed in SRT-Server to improve the speed of internet connection while maintaining the privacy of the client’s IP address. This framework allows for efficient and effective data transfer between the client and server while ensuring the confidentiality and security of user data. The proxy client in SRT-Server acts as an intermediary between the client and server, intercepting requests from the client and forwarding them to the server. This setup ensures that the client’s IP address remains hidden from the server, protecting the client’s privacy and confidentiality. In addition, the proxy client also helps to improve the speed of internet connection by caching frequently accessed data, reducing the need for repeated requests to the server. This setup improves the overall efficiency of SRT-Server, making it a reliable and user-friendly tool for SRT data analysis.

### Analytic modules on SRT-Server

Here, we provide details for the input data format and the ten analytic modules deployed on SRT-Server. Some modules also contain a set of reference datasets deposited on the SRT-Server that are required by certain analytic functions inside these modules.

#### Input format

SRT-Server supports a generic data input format as well as four platform-specific data formats for SRT data collected from four common SRT platforms: 10 × Visium, VIZGEN MERFISH, SeqFISH, and Slide-seqV2. The generic data format consists of two matrices: a gene expression matrix containing gene expression counts across genes (rows) for measured spatial locations (columns), and a location information matrix containing the *x* and *y* coordinates (columns) across measured spatial locations (rows).

For platform-specific data formats, SRT-Server accepts output files from the aforementioned common SRT platforms. For 10 × Visium data [5], users can upload data output from Space Ranger in the form of an h5 format gene expression matrix, along with an additional file folder containing H&E staining images and location information. For MERFISH data [8], users can upload a file with a name ending in “cell_by_gene.csv” for gene expression and a file with a name ending in “cell_metadata.csv” for meta data. For Slide-seqV2 data [6], users can upload a file with a name including the phrase “expression” for gene expression and a file with a name including the phrase “location” for location information. For SeqFISH data [9], users can provide three file folders: (i) a cell_locations folder containing at least three files named “centroids_annot.txt,” “centroids_coord.txt,” and “centroids_offset.txt;” (ii) a count_matrix folder containing at least one file named “expression.txt;” and (iii) a raw_data folder containing at least one file named “location_fields.png.” This flexibility in data input formats allows users to easily upload their SRT data to SRT-Server regardless of the SRT platform used, making it a versatile and user-friendly tool for SRT data analysis.

#### Module for quality control (QC)

This module performs standard quality control for SRT datasets. The QC module utilizes two functions (*Load10X_Spatial* and *CreateSeuratObject*) from the Seurat package to initially create a Seurat object, either for the 10 × Visium data format or for other data formats. Standard QC is then conducted using *min.features* (default setting: 100) and *min.cells* (default setting: 100) parameters. *min.features* is used to filter out measured locations with a low number of expressed genes, while *min.cells* excludes features with a low number of expressed spots. In addition to these standard filtering processes, the QC module carries out extra gene filtering on data from the Visium and MERFISH platforms. Specifically, for Visium, SRT-Server removes the non-coding transcripts, such as microRNA (miRNA) and long non-coding RNA (lncRNA), which are not tested on other platforms. For MERFISH, SRT-Server removes genes with names starting with “Blank”. After gene and location filtering, the QC module saves the processed SRT data in the h5 format, which contains five information groups: gene expression matrix, location information, spot label, gene ID, and platform information. If multiple SRT samples are uploaded, the QC module applies the same parameter settings to perform QC for all samples, obtains the intersection of retained genes across samples, and saves the processed data into h5 format groups. The h5 format output from the QC module is compatible with various software programs, including R and python. Specifically, when using the QC module, user first drags *Input Data* and *QC* module to the canvas, connect them, and click the *Save* button. Afterwards, user can click *Input Data* to select or upload SRT data for analysis and can also set up parameter arguments for the *QC* module.

#### Module for identifying SVGs (SVG)

This module performs analysis to identify SVGs. It includes two different SVG detection methods: SPARK [19] and SPARK-X [20] as well as two standard spatial statistics tests Moran’s I and Geary’s C. SPARK employs a generalized linear spatial model (GLSM) with different kernel functions to directly model count data from SRT. It also uses an efficient penalized quasi-likelihood (PQL) algorithm for model fitting and an exact formula to test the spatial variance component for calibrated SVG detection. SPARK-X is a non-parametric version of SPARK and is highly scalable to very large-scale SRT datasets. SPARK can be more powerful than SPARK-X on datasets where SPARK is scalable, while SPARK-X is much more computationally efficient than SPARK so that it can handle extremely large-scale SRT datasets that SPARK cannot. In addition to these methods, the SVG module also offers a set of plotting functions (SVG_Plot) capable of producing three types of plots to visualize SVG analysis results. These plots include a qqplot that displays the distribution of SVG detection *P* values, a location feature plot that showcases the top significant SVGs, and a pattern plot that reveals the spatial expression patterns of the detected SVGs.

#### Module for cell type deconvolution (DECON and DECON_PY)

This module performs cell type deconvolution at each measured spatial location for spot level SRT datasets, such as 10 × Visium. The DECON module employs three methods, including CARD implemented in the DECON module, as well as cell2location and Tangram implemented in the python version of the DECON module (DECON_PY), for deconvolution analysis. CARD is a computationally efficient method that relies on a non-negative matrix factorization to perform deconvolution while using a conditional autoregressive (CAR) modeling assumption to encourage similarity in cell type composition in neighboring spatial locations, thus optimizing deconvolution performance [26]. CARD can perform either reference-based deconvolution or reference-free deconvolution. The former uses an external scRNA-seq dataset measured on the same tissue as a reference, while the latter only requires the name of a list of cell marker genes for the target tissue. cell2location uses an approximate variation inference in the scvi-tools framework for deconvolution [35]. Tangram uses a nonconvex optimization algorithm to estimate the probabilistic mapping matrix between a scRNA-seq data and the SRT data [36]. The cell mode of Tangram requires the scRNA-seq and SRT data collected from the same sample, while the cluster mode of Tangram supports different samples for the two datasets. For the reference-based version of CARD, cell2location, and Tangram, SRT-Server provides 51 scRNA-seq data to serve as reference. For the reference-free version of CARD, SRT-Server also provides gene marker sets from 20 normal tissue datasets in mice [37] and 8 normal tissues with 23 diseases status in humans (Additional file 1: Table S1 and S2). Moreover, we have added an Upload Selection option for the user to first choose whether to upload their own reference data or not. In the case of user choosing to upload their own reference data, a cascade will show up, displaying all the files in the current data space with options for users to upload their own reference file. In addition to deconvolution analysis, the DECON module also offers a set of plotting functions (DECON_Plot) to produce four types of plots to visualize deconvolution results. These plots include a pie plot that displays the proportion of different cell types on each measured location, a cell type proportion plot that displays the proportion of a specific cell type on each location, a feature plot that displays expression measurements for a given gene across locations, and a cell type location plot that displays the location of different cell types. Some of the plots can be created at enhanced resolution thanks to the ability of CARD in creating refined spatial maps with a resolution higher than that measured in the original study.

#### Module for spatial domains detection (SDD)

This module performs spatial clustering to group spatial locations into distinct tissue domains in a spatially informed manner. The SDD module employs two different methods for spatial clustering: (1) *SDD_sPCA*: the spatially aware principal component analysis (SpatialPCA); and (2) *CL_jo*: the Bayesian analytics for spatial segmentation (BASS). SpatialPCA first performs spatial PCA to extract the spatial PCs, which represent a low-dimensional embedding for the gene expression matrix with spatial localization information. Afterwards, SpatialPCA performs either *k*-means clustering or Louvain clustering on the extracted spatial PCs. Users can choose the desired number of spatial domains during the analysis. When handling multiple SRT samples, SpatialPCA in the SDD module directly uses the standard integration pipeline in Seurat to integrate gene expression data across multiple samples before preforming spatial PCA and spatial domain detection. BASS is a Bayesian hierarchical model that carries out multi-scale analysis in the form of cell type clustering and spatial domain detection. Users can similarly input the desired number of spatial domains and the desired number of cell types to BASS during the analysis. When handling multiple SRT samples, BASS in the SDD module supports two analysis options: *merge* [30], which directly merges the raw count matrices from different samples; and *harmony* [38, 39], which iteratively removes batch effects from different samples using the harmony approach.

In addition to analytic methods, the SDD module also offers a set of plotting functions that can generate two types of plots. These plots include a location plot that displays the spatial clustering results across spatial locations and a heatmap that displays the average expression levels for a set of genes (either the top 50 highly variable genes or a set of genes chosen by user) across the detected spatial domain clusters. Importantly, the SDD module provides an interactive page, which contains multiple interactive panels. Specifically, the first panel contains a location plot, which displays the inferred spatial domain labels for the spatial locations. These labels are colored with the default color scheme. The second panel shows the same location plot but allows users to choose their own color scheme. The third panel contains a spatial expression plot, which displays gene expression level across tissue locations. The fourth panel contains the same location plot with the selected spatial domains. Users have the option to select which gene to display on the third panel and which spatial domain to display on the fourth panel. Besides these four panels, the SDD module also provides a table to display the corresponding DEG information, including gene ID, cluster number, *P*-value, and FDR.

#### Module for cell typing (CT)

This module supports two distinct analyses for single-cell resolution SRT data, such as MERFISH: (1) cell typing (*CT_PCA* and *CL_jo*), also known as cell type clustering, which infers cell types and clusters each cell into a cell type; and (2) cell type annotation (*CT_Annot*), where the inferred cell types are annotated based on a reference panel or known cell type marker genes. The module provides two cell typing methods: principal component analysis (PCA) and joint model. The PCA method implements the standard Seurat pipeline that applies PCA to perform dimensional reduction, extracts the top PCs that explain at least 80% of gene expression variance, and conducts cell type clustering based on the top PCs using the *k*-means clustering algorithm. The PCA method allows users to specify a resolution parameter to achieve the desired number of cell type clusters. When handling multiple SRT samples, the PCA method in the CT module offers three options for data harmonization: *merge*, *integration* [30], and *SCTransform* [40]. The *merge* option is consistent with what has been explained in the previous module. Both *integration* and *SCTransform* options perform data normalization in each dataset first before harmonizing them across datasets. Specifically, the *integration* option normalizes the gene counts for each cell by dividing with the total counts for the cell, multiplying a scaling factor of 10,000, and applying the natural logarithm transformation. The *SCTransform* option uses a modeling framework for variance stabilization and obtains Pearson residuals from regularized negative binomial regression. Subsequently, both *integration* and *SCTransform* options use the canonical correlation analysis (CCA) to project the normalized data into a subspace and identify the mutual nearest neighbors in the CCA subspace to serve as anchors for data harmonization. For both *integration* and *SCTrasform* options, users can freely set the number of anchors and the number of canonical correlation vectors in the data harmonization step.

In addition to cell typing, SRT-Server can also perform cell type annotation using two approaches: scSorter [24] and Garnett [25]. scSorter requires an additional input file containing a list of cell type-specific marker genes. It first employs a likelihood function to integrate the expression levels of marker genes with that of non-marker but highly variable genes, then optimizes the likelihood function to annotate known and unknown cell types. Garnett also requires a file that contains a list of cell type-specific marker genes, in addition to the SRT data. Garnett first selects “representative” cells using marker genes for each cell type, trains a multinomial classifier using an elastic net regression on the “representative” cells using all genes, and then classifies the “non-representative” cells using the trained classifier. In addition, like the DECON module, user can also upload their own reference scRNA-seq data.

Aside from these analytic methods, the CT module also includes a set of plotting functions that can generate three types of plots. These plots include a location plot that displays the cell type clustering results across spatial locations, a scatter plot that displays the low-dimensional embedding based on the Uniform Manifold Approximation and Projection (UMAP), and a heatmap that displays the average expression levels for a set of genes (either the top 50 highly variable genes or genes chosen by user) across the cell type clusters. Importantly, the CT module provides an interactive page, which contains multiple interactive panels. Specifically, the first panel contains a location plot, which displays the inferred cell type labels. These labels are colored with the default color scheme. The second panel shows the same location plot but allows users to choose their own color scheme. The third panel contains a spatial expression plot, which displays gene expression level across tissue locations. The fourth panel contains the same location plot with the selected cell types. Users have the option to select which gene to display on the third panel and which cell type to display on the fourth panel. Besides these four panels, the CT module also provides a table to display the corresponding DEG information, including gene ID, cluster number, *P*-value, and FDR.

#### Module for detecting DEGs (DEG)

This module aims to identify genes that are differentially expressed either within a specific spatial domain, utilizing output from the SDD module, or within a specific cell type, using output from the CT module. Following the recommendations of [31], the DEG module implements the Wilcoxon rank-sum test from the *FindAllMarkers* function in Seurat to detect DEGs. In addition to the DE analysis method, the DE module also includes a set of functions that generate two types of plots to visualize DE results. These plots include a heatmap that displays the gene expression levels for a set of selected genes across clusters, and a feature plot that displays the expression level of the specific genes across locations.

#### Module for identifying pathways that are enriched with DEGs or SVGs (ORA)

This module aims to identify biological pathways that are enriched in the DEGs detected by the DEG module or in the SVGs detected by the SVG module. The ORA module implements the ORA function in clusterProfiler or ReactomePA for pathway enrichment analysis and includes five pathway databases: Gene Ontology (GO) [41], Kyoto Encyclopedia of Genes and Genomes (KEGG) [42], WikiPathways [43], ReactomePA [44], and Disease Ontology (DO) [45]. Dependent on the species of the data, the ORA module converts the gene symbols to Ensembl ID using the *bitr* function either in org.HS.eg.db for human [46] or in the org.Mm.eg.db for mice [47]. In addition to pathway enrichment analysis, the ORA module also offers a set of plotting functions for generating two types of plots to visualize the results. These plots include a bubble plot that displays the detected enriched biological pathways, and a dot plot that displays *P* value and the ratio for the top ten significant pathways.

#### Module for detecting cell–cell or domain-domain communications (CCC)

This module aims to detect either cell–cell communications based on the output from the CT module or domain-domain communications based on the output from the SDD module. Using the results from CT or SDD, the CCC module employs SpaTalk and cellchat to detect communications through ligand-receptor (LR) interactions [29, 48]. The CCC module offers a total of 20 LR databases in mouse and human: one from SpaTalk.DB [29] and 18 from the LIANA databases (i.e. iTALK, CellTalkDB, CellPhenoDB, and so on) [28], and one from gitto.mouse [13]. With a user-selected LR database, SpaTalk constructs a cell graph network using the K Nearest Neighborhood (KNN) algorithm, counts the 1-hop neighbor nodes of receivers for each sender using the LR database, and defines the significant communications by shuffling cell labels to recalculate the number of LR interaction pairs. Based on cellchatdb, cellchat estimates the LR strength and tests its significance using permutation test.

In addition to detecting communications, the CCC module also offers a set of plotting functions to generate two types of plots for visualizing the communication detection results [48]. These plots include a circle plot that displays the number of significant LR pairs between cell type pairs or spatial domain pairs, and a dot plot that displays the magnitude for a selected set of LR pairs between cell type pairs or spatial domain pairs. Importantly, the CCC modules provides an interactive page, which contains a pathway information table, a circle plot connecting the identified interactions among cell types or spatial domains, a cell–cell communication network plot displaying the magnitude and significance of selected cell type pairs, and a heatmap to visualize the interaction strength and significance for pairs of genes from a gene pathway that is chosen by the user.

#### Module for estimating the trajectory across cells or across spatial locations (TRAJ)

This module aims to estimate the pseudo-time trajectory either across spatial locations or across cells. The pseudo-time trajectory inference is straightforward on single-cell resolution SRT data. However, it can become challenging and difficult to interpret when directly applied to the gene expression measurements collected in non-single-cell/spot resolution SRT data. In particular, in the spot resolution SRT data, each measured spot may contain a mixture of cells from potentially heterogeneous cell types. Consequently, the pseudo-time inferred on each spot likely represents the average pseudo-time across the cells on the spot, making the interpretation challenging. Therefore, for single-cell resolution spatial transcriptomics, TRAJ can directly take as input the low dimensional components output from the PCA method in the CT module to perform trajectory inference across cells on the tissue. In addition, TRAJ can take as input the low-dimensional components output from the SpatialPCA method in the SDD module to perform trajectory inference across tissue locations. For spot resolution spatial transcriptomics, TRAJ can take as input the cell type-specific expression estimates output from the three methods in the DECON module to construct pseudo-time across cells within a particular cell type to capture the developmental stages of the cells in the cell type. In either case, the TRAJ module employs Slingshot to perform trajectory analysis across cells or spatial locations. Slingshot uses a cluster-based minimum spanning tree to stably identify the key elements of the global lineage structure. Then, Slingshot uses simultaneous principal curves to fit smooth branching curves. In addition to trajectory analysis, the TRAJ module also offers a set of plotting functions that can produce three types of plots to visualize the trajectory inference results. These plots include a scatter plot that displays how gene expression changes over pseudo time for selected gene, a heatmap that shows the expression levels of the top 50 genes associated with the inferred pseudo-time, and a location plot to visualize the inferred pseudo-time across spatial locations on the tissue.

#### Module compatibility

We note that while we have described each of the ten analytic modules in the SRT-Server separately, it is important to note that there is a partial hierarchical structure among these modules (Fig. 1). Specifically, the QC module is typically positioned at the beginning of the analysis pipeline before any other modules. The DE, CCC, and TRAJ modules all require output from either the SDD module or the CT module. The ORA module requires output from either the SVG module or the DEG module. Finally, the DECON and DECON_PY modules are specifically designed for spot resolution SRT datasets while the CT module is specifically designed for single-cell resolution SRT datasets.

### Datasets for case studies

We demonstrate the utility of the SRT-Server through three case studies involving SRT datasets collected by various technologies and on distinct tissues and species. In Case Study 1, we obtained the 10X Visium dataset collected on mouse brain sagittal anterior sections from the 10X Visium spatial gene-expression repository (https://www.10xgenomics.com/resources/datasets/mouse-brain-serial-section-2-sagittal-anterior-1-standard). The data contains 19,330 transcripts measured on 2695 spots. In Case Study 2, we obtained the 10X Visium dataset collected on two tissue samples of HER2 + breast cancer (samples GSM5732357 and GSM5732358 from GSE190811) [10]. The data contains 14,511 transcripts measured on 3055 and 4454 spots in the two samples, respectively. In Case Study 3, we acquired the Vizgen dataset collected on the mouse brain (slice 2 replication 3; https://info.vizgen.com/mouse-brain-data) and selected a quarter of the slice centering on the hippocampus for analysis. The analyzed hippocampus data contains 483 genes measured on 20,100 cells.

## Results

### Overview of SRT-Server

The SRT-Server is a user-friendly, highly expandable server framework designed to facilitate the analysis of SRT studies (Fig. 1). A comprehensive description of the SRT-Server is provided in the “Methods” section. In summary, the SRT-Server enables users to construct an entire SRT analysis pipeline without any prior programming knowledge by utilizing ten analytic modular building blocks. Each analytic module serves a specific SRT analytic task, such as quality control, SVG detection, cell type deconvolution for spot resolution SRT, clustering (cell type clustering for single-cell resolution SRT studies or spatial domain detection on the tissue), DEG detection in specific cell types or spatial domains, pathway enrichment analysis, cell–cell communication detection with spatial location information, and pseudo-time trajectory inference with dimensional reduction results. Users can simply drag these modules onto an interactive canvas within the SRT-Server and connect them to form customized analytic pipelines. Furthermore, the SRT-Server’s framework is highly adaptable, capable of incorporating new analyses and modules as they emerge, provided they are supported by an R package and adhere to pre-specified dependency structures (Fig. 2). The codeless environment and intuitive interface of the SRT-Server prove invaluable for biologists, allowing them to perform thorough and rigorous SRT analyses without requiring a local computational environment, programming language proficiency, or prior statistical and computational knowledge. We demonstrate the advantages of the SRT-Server through three case studies.

### Case Study 1: 10X Visium Data on Mouse Brain

**Fig. 3:**
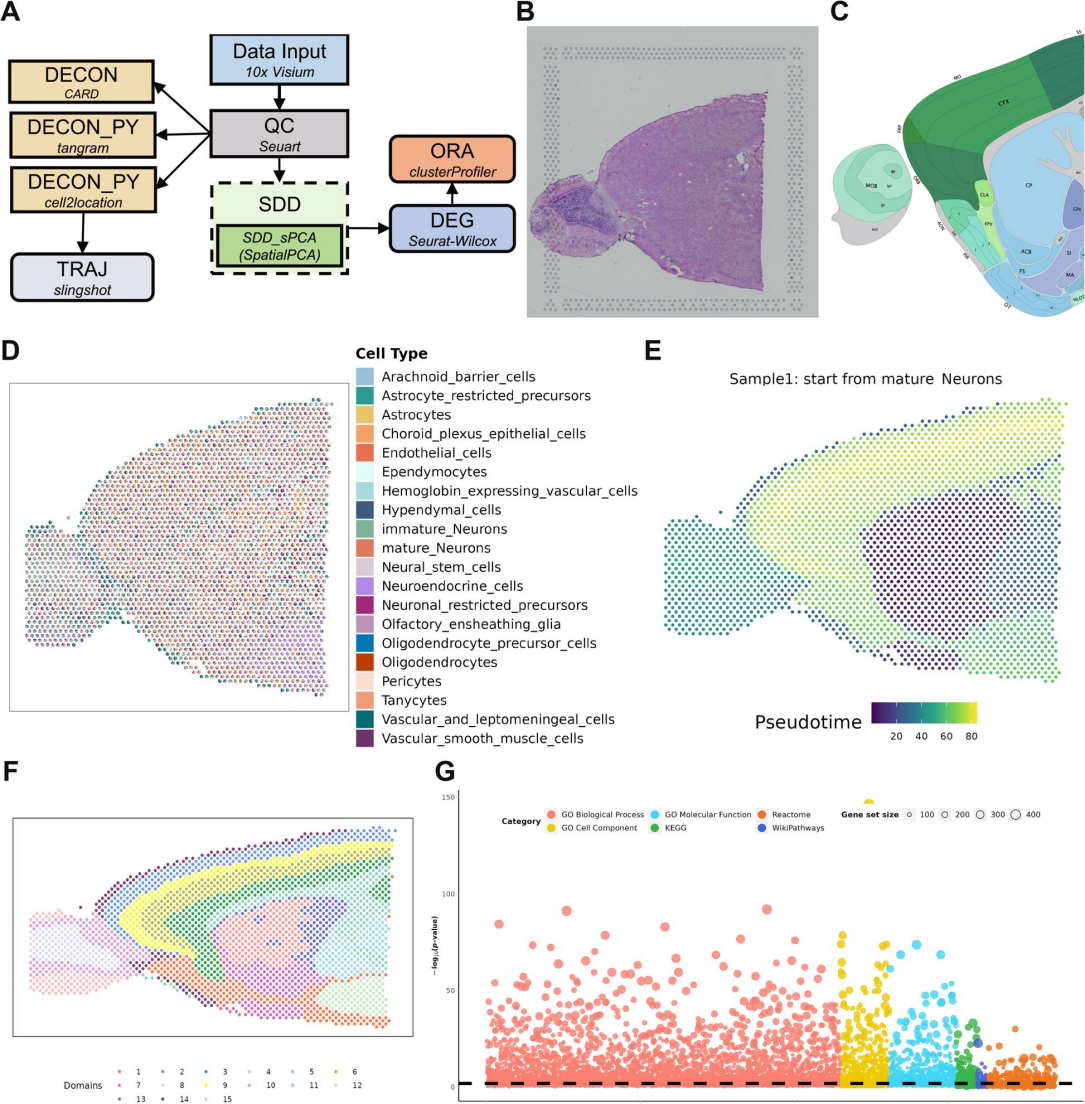
Analytic pipeline and results summary for the anterior mouse brain 10X Visium dataset. **A** The analytic pipeline built on the SRT-Server. The constructed analytic pipeline uses eight analytic modules, with the parameter settings for each module visualized on the panel. **B** The H&E staining image displays the general tissue structure of the anterior mouse brain. **C** A corresponding annotated brain tissue section from the Allen Brain Atlas. **D** A spatial scatter pie plot displays the inferred cell-type compositions across spatial locations from CARD in the DECON module. **E** The trajectory inferred by mature neuron whose proportion is estimated by cell2loction. **F** Fifteen detected spatial domains from SpatialPCA in the SDD module. **G** Bubble plot displays − log10(*p*-values) for different pathways from the ORA module (*y*-axis). Six pathways are colored: GO biological process (red), GO molecular function (yellow), GO cellular component (blue), KEGG (grass-green), Reactome (grass-green), and Wikipathways (purple)

We first examined a 10X Visium dataset collected from sagittal sections of the anterior region of the mouse brain. To analyze the data, we constructed an analytic pipeline on SRT-Server by dragging and connecting six analytic modules (QC, DECON, DECON_PY, TRAJ, SDD, DEG, and ORA; Fig. 3A, Additional file 1: Table S3, and Additional file 3: Fig. S1). This pipeline enabled us to perform quality control, spatial domain detection using SpatialPCA, cell type deconvolution with CARD, cell2location and tangram, pseudo-time trajectory inference using slingshot, DEG detection through the Wilcoxon test, and ultimately, pathway enrichment analysis for the identified DEGs. The hematoxylin and eosin (H&E) stained image of the tissue structure, along with its anatomical annotations in sagittal sections from the Allen Brain Atlas [49], are provided in Fig. 3B, C.

After performing QC using default settings, we obtained data for 12,528 transcripts measured across 2695 spots. We first conducted reference-based CARD with the DECON module, utilizing a mouse brain scRNA-seq dataset hosted on SRT-Server as the reference. The reference scRNA-seq dataset comprises expression measurements for 14,699 genes across 32,621 cells belonging to 20 cell types (Additional file 3: Fig. S2) [50]. The DECON analysis accurately estimated the expected layered structure of the mouse brain (Fig. 3D and Additional file 3: Fig. S3-S4). Some key findings in the DECON analysis include mature neurons, on average, represented the highest cell type proportion across spots (mean = 63.23%, median = 71.03%); the proportion of olfactory ensheathing glia was significantly higher in the olfactory bulb (OB) than that in other tissue domains (*t* = 8.26, *P* value = 6.63E − 13); astrocyte proportion was higher in the first layer of the OB or cerebral cortex than in the other tissue domains (*t* = 25.02, *P* value = 2.01E − 68). In addition, we compared three different deconvolution methods, including CARD, cell2location, and Tangram, in the DECON module. First, we found that the deconvolution results between CARD and cell2location are highly consistent (*R* = 0.949, *P*-value = 0.00E + 00, 95%CI 0.948–0.950), while the results from Tangram are different from the other two methods (Additional file 3: Fig. S5). Next, we carefully examined the devolution results on individual cell types and found that the key findings from cell2location are again consistent with those of CARD, both of which are different from Tangram. Specifically, as expected, both CARD and cell2locaton correctly identified the mature neurons to be the most abundant cell type in the brain (estimated mean proportion for CARD = 63.23%; estimated mean proportion for cell2location = 54.94%), while Tangram identified mature neuron to be the fourth abundant cell type (mean proportion = 6.32%). In addition, both CARD and cell2location estimated astrocytes to be enriched in the first layer of the OB or in the cerebral cortex as compared to the other parts of the brain (*t* = 25.02, *P* value = 2.01E − 68 for CARD;* t* = 15.94, *P* value = 2.56E − 39 for cell2location) while Tangram did not (*t* =  − 0.06, *P* value = 0.95). Finally, we found CARD to be much more computationally efficient than the other two methods. The computation time for CARD, cell2location, and Tangram are 0.092, 10.968, and 0.267 h, respectively.

In parallel to cell type deconvolution with DECON, the constructed analysis pipeline on SRT-Server also carried out spatial domain detection analysis using the TRAJ module. We used cell2location for deconvolution and obtaining the cell type-specific expression information, with which we further estimated the pseudo-time trajectory using slingshot in the mature neurons (Fig. 3E). The inferred neuronal trajectory captures the key features of the rostral migratory stream, a migration path of newly generated neuroblasts that migrate from the sub ventricular zone of the lateral ventricles into the OB [51]. In particular, the pseudo-time of OB (domain 8) is significantly higher than that of the lateral ventricle (domain 9) (*t* = 117.11, *P*-value = 5.38E − 183).

In parallel to cell type deconvolution with DECON, the constructed analysis pipeline on SRT-Server also carried out spatial domain detection analysis using the SDD module. Here, we selected the SpatialPCA method in SDD and set the domain number to be 15. We also set the number of domains to be 10 or 20 to explore domain detection results under different pre-specified domain numbers (Additional file 2: Fig. S6). In SDD, we employed the Gaussian kernel function to construct the distance kernel matrix across spots, used the Walktrap method for clustering spatial domains, and applied SPARK-X to select the top 3000 SVGs genes for dimensionality reduction (Fig. 3F). The detected spatial domains captured the three main anatomic structures of the mouse brain, including the cerebral cortex (e.g., olfactory area, somatomotor area, orbital area, and piriform area), the cerebral nuclei (e.g., caudoputamen, nucleus accumbens, and olfactory tubercle), and fiber tracts. Each spatial domain consists of a distinct cell type composition and each cell type often displays enrichment in specific domains. For example, the proportion of astrocytes is higher in the cortex layer 1 (domain 14; 21.40%) than in the remaining domains (*t* = 18.86, *P* value = 9.92E − 38). The location plot based on using 15 spatial domains was more consistent with both anatomical annotations and DECON results than those from 10 and 20 domains.

The constructed analysis pipeline further performed DE analysis and detected a total of 26,932 domain-specific DEGs across 15 spatial domains, with an average of 1796 DEGs per domain (ranging from 22 to 4986; FDR < 0.01). Many of the detected DEGs are known cell type marker genes (Fig. 3D, E and Additional file 2: Fig. S7). For example, *Plp1* is a known marker gene for oligodendrocytes and is involved in axon-supportive function of myelin [52, 53]. It is detected as a domain-specific DEG for domain 11 (fiber tracts; *log*_*2*_*FC* = 1.67, FDR = 6.15E − 66), which contains a much higher proportion of oligodendrocytes than the other domains (*t* = 20.10, *P* value = 4.95E − 52). As another example, *Omp* is involved in signal transduction and odor discrimination while *S100a5* is a brain-specific calcium-binding protein [54–57]. Both are detected as domain-specific DEGs for domain 15 (glomerular; *Omp*, *log*_*2*_*FC* = 1.35, FDR = 1.96E − 254; *S100a5*, *log*_*2*_*FC* = 4.61, FDR = 2.07E − 128), where the proportion of olfactory ensheathing glia is much higher than the other domains (*t* = 8.26, *P* value = 6.63E − 13). As a third example, *Igfbp2* is known to be enriched in astrocytes in gray matter [58]. It is detected as domain-specific DEGs for domain 5 (cortical layer 1; *log*_*2*_*FC* = 0.75, FDR = 1.16E − 22), where the proportion of astrocytes is higher than the other domains (*t* = 11.07, *P* value = 4.82E − 22).

With the detected DEGs, the constructed analysis pipeline performed ORA using six databases: GO Biological Process, GO Cell Component, GO Molecular Function, KEGG, Reactome, and Wikipathway. We first obtained significant pathways using all DEGs and a total of 5247 significant pathways (FDR < 0.01) were detected. Examples include regulation of synapse organization (*P* value = 1.53E − 70), dendrite development (*P* value = 4.45E − 74), and regulation of neurogenesis (*P* value = 7.82E − 64) (Fig. 3G). We then obtained domain-specific significant pathways using domain-specific DEGs and detected 21,049 significant domain-specific pathways based on the same FDR criterion, with, on average, 1403 significant pathways per domain (median = 1248, ranging from 40 to 3150). For example, 42 significant pathways related to synapse, such as synapse organization (*P* value = 6.24E − 41), were detected for domain 3 [59].

### Case study 2: 10 × Visium Data on HER2 + Breast Cancer

**Fig. 4:**
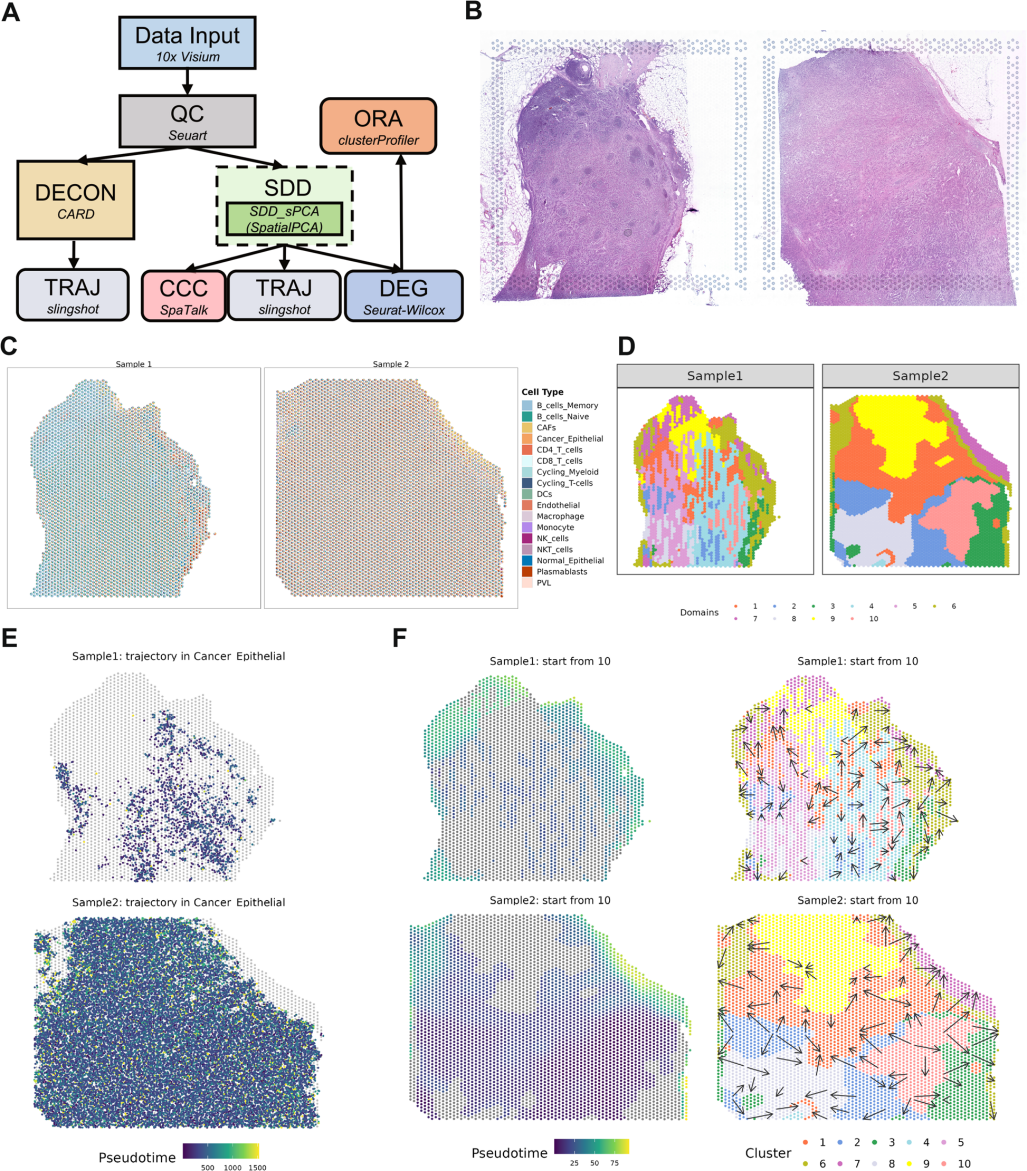
Analytic pipeline and results summary for two tissues from the HER2 + BRCA 10 × Visium dataset. **A** The analytic pipeline built on the SRT-Server. The constructed analytic pipeline uses seven analytic modules, with the parameter settings for each module visualized on the panel. **B** The H&E staining image displays the general tissue structure. **C** A spatial scatter pie plot displays the inferred cell-type compositions across spatial locations from CARD in the DECON module. **D** 10 detected spatial domains from SpatialPCA in the SDD module. We used the clustering result in domain number = 10 to all the downstream analysis, which is not mentioned in the pipeline. **E** The pseudo-time inference result in the cancer epithelial is obtained from slingshot in the TRAJ module after DECON analysis. **F** The pseudo-time inference results are obtained from slingshot in the TRAJ module. The left two panels show the pseudo-time of different cell types on the two samples. The right two plots include arrows to display pseudo-time change on the tissue: the arrow point from tissue locations with low pseudo-time towards tissue locations with high pseudo-time

Next, we analyzed a 10X Visium data collected on lymph node metastasis (LNM) from four breast cancer patients using SRT-Server [10]. For analysis, we built an analytic pipeline by dragging and connecting seven analytic modules on the server (QC, DECON, SDD, DEG, ORA, TRAJ, and CCC; Fig. 4A, Additional file 1: Table S3, and Additional file 2: Fig. S8). The built pipeline allows us to perform quality control, spatial domain detection with SpatialPCA, cell type deconvolution with CARD, pseudo-time inference with slingshot, DEG detection with Wilcox test, pathway enrichment analysis on the detected DEGs, cell–cell communication identification using SpaTalk, and pseudo-time trajectory inference using slingshot. The H&E image of the tissue structure is provided in Fig. 4B.

After QC, we obtained expression measurements for 9817 genes for the two HER2 + samples, with 3051 and 4430 cells, respectively. We first performed cell type deconvolution using the DECON module, where we selected CARD for analysis with the reference data of HER2 + BRCA scRNA-seq that consists of 29,733 transcripts on 19,311 cells from 17 cell types (Additional file 2: Fig. S9) [60]. The estimated cell type proportions on the SRT data are displayed in Fig. 4C and Additional file 2: Fig. S10. We found that the composition of different cell types varies across the two HER2 + samples, suggesting tumor heterogeneity in breast cancer [61]. For example, cancer epithelial cells were the fourth most abundant cell type in the first sample (mean = 7.59%, median = 8.51%) but were the most abundant cell type in the second HER2 + sample (mean proportion = 13.81%, median = 14.32%). The proportion of memory B cells and naïve B cells was higher in the first sample than the second sample (*P* value for memory B cells < 2.2E − 16; *P* value for naïve B cells < 2.2E − 16), while the proportion of cancer-associated fibroblasts (CAF) is higher in the second sample than the first sample (*t* = 5.71, *P* value = 1.18E − 08). In addition, based on the deconvolution results from CARD, we estimated the pseudo-time in the cancer epithelial cells using TRAJ module and identified 148 genes to be associated with epithelial-mesenchymal transition (EMT) based on an FDR threshold of 0.05 (Fig. 4E, Additional file 2: Table S4) [62].

In parallel to cell type deconvolution with DECON, the constructed analysis pipeline on SRT-Server also carried out spatial domain detection analysis using the SDD module, with the same parameter settings as in Case Study 1 (Fig. 4D and Additional file 2: Fig. S11). The location plot based on using 10 spatial domains was more consistent with DECON results than those from 15 and 20 domains. The detected spatial domains, when paired with further trajectory inference, reveal gradient changes from the tumor towards tumor-adjacent regions (Fig. 4F and Additional file 2: Fig. S12). We also combined the results from DECON with that from SDD and TRAJ and identified several cell types that are enriched in certain spatial domains. Specifically, first, for the second sample, the proportion of CAFs is higher in domain 7 than the other domains (mean in domain 7 = 33.26%, mean in other domain = 4.80%, *t* = 17.04, *P* value = 9.53E − 37). For the first sample, there is no significant difference in CAFs detected between these domains (*t* = 1.76, *P* value = 0.08). Based on evidence that inflammatory-like CAFs (iCAFs) disperse across invasive cancer region, we speculate that CAFs in the second sample might be iCAFs [60, 63]. Accordingly, *S100A4* (*log*_*2*_*FC* = 0.37, FDR = 6.50E − 2), which classifies CAF into iCAFs and non-iCAFs, is one of the DEGs for domain 7 [64, 65]. Second, the proportion of cancer epithelial cells is higher in domain 10 than in other domains for both samples (Sample 1: mean in domain 10 = 11.00%, mean in other domains = 7.45%, *t* = 15.15, *P* value = 2.96E − 32; Sample 2: mean in domain 10 = 16.97%, mean in other domains = 13.42%, *t* = 45.46, *P* value < 1.05E-267). *PTPRT* (*log*_*2*_*FC* = 1.23, FDR = 3.74E − 153), which plays an important role in cell–cell adhesion and has mutational inactivation of its phosphatase, is the top DEG in domain 10 [66]. In addition, the pseudo-time inferred on domain 7 is statistically significantly later than that of domain 10 (*t* = 84.19, *P* value = 0.00E + 00), which has a higher percentage of CAF. With the detected spatial domains, the constructed analysis pipeline performed DE analysis and detected a total of 23,547 domain-specific DEGs across 10 domains, with 2354 DEGs per domain (ranges from 5 to 4746; FDR < 0.01) (Additional file 2: Fig. S13). For example, *FAM129C* (*log*_*2*_*FC* = 1.58, FDR = 1.80E − 45) is one of DEGs for domain 7 and also one of the marker genes for B memory cells [67]. With the detected DEGs, we performed ORA and detected 1818 significant pathways with FDR < 0.01. Example significant pathways include 37 pathways associated with immune response and immune regulation and 27 pathways associated with cancer (Additional file 2: Fig. S14).

Finally, with the detected domains and the detected cell proportions on each spot, the constructed analysis pipeline performed CCC analysis using SpaTalk, with SpaTalk.DB serving as the LR database (Fig. 4F and Additional file 2: Fig. S15). In the CCC analysis, based on a *P* value threshold of 0.01, we detected 10,165 significant ligand-receptor pairs across cell types (ranges from 31 pairs for NKT cells to 1809 for CAF). For example, the ligand *EGF* in cancer epithelial cells is significantly associated with the receptors *ERBB2* (*P* value = 0.009) and *EGFR* (*P* value = 0) in CAFs, suggesting a potential signaling cascade from the former to the latter. The two receptors belong to the ErbB family which can be activated by eight ligands including *EGF*. *EGFR* is expressed in almost all nonneoplastic cell types in TME, including CAFs [68].

### Case Study 3: MERFISH Data on Mouse Brain

**Fig. 5:**
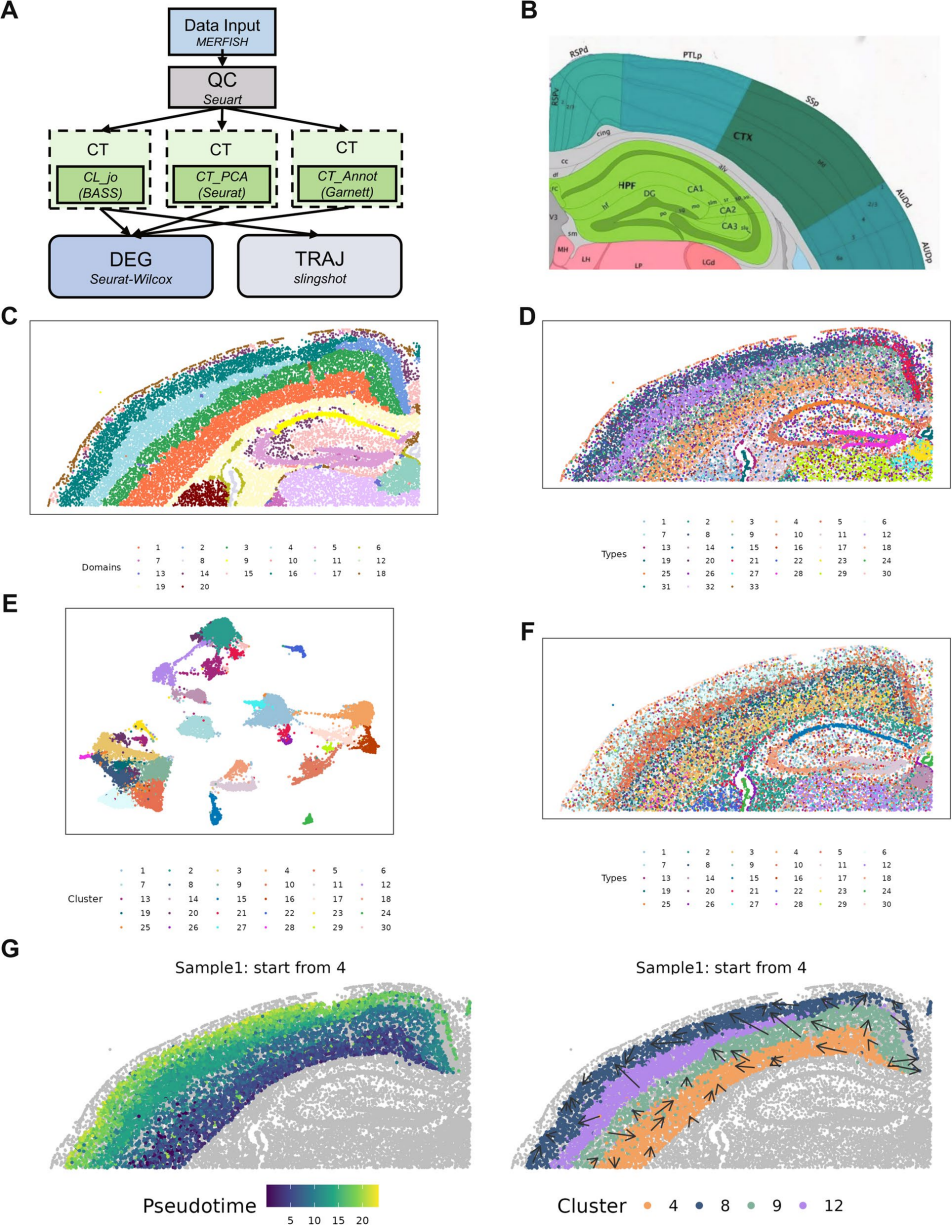
Analytic pipeline and results summary for analyzing the mouse brain MERFISH dataset. **A** The analytic pipeline built on the SRT-Server. The constructed analytic pipeline uses five analytic modules, with the parameter settings for each module visualized on the panel. **B** A corresponding annotated brain tissue section from the Allen Brain Atlas. **C** 20 detected spatial domains from BASS in the CL_jo module. **D** 40 detected cell types from BASS in the CL_jo module. **E** The UMAP plot for 30 detected cell types from Seurat in the CT_PCA module. **F** Thirty detected cell types from Seurat in the CT_PCA module with resolution = 1. **G** The pseudo-time inference results are obtained from slingshot in the TRAJ module. The left panel shows the pseudo-time of four selected cell types. The right two plots include arrows to display pseudo-time change on the mouse brain: the arrow point from tissue locations with low pseudo-time towards tissue locations with high pseudo-time

Finally, we examine a MERFISH data collected from mouse brain. For analysis, we built an analytic pipeline by dragging and connecting six analytic modules (QC, CL_jo, CT_annot, CT_PCA, TRAJ, and DEG; Fig. 5A, Additional file 1: Table S3, and Additional file 2: Fig. S16). The built pipeline allows us to perform quality control, joint cell type and spatial domain detection with BASS, cell type clustering with Seurat, cell type classification with Garnett by external scRNA-seq reference panel, pseudo-time inference with slingshot, and DEG detection with Wilcox test. The anatomic annotations for the tissue structures based on Allen Brain Atlas [49] are provided in Fig. 5B.

After quality control, we obtained 483 genes and 20,100 cells. Because the data is of single-cell resolution, we performed both spatial domain detection and cell type clustering using the multi-scale method BASS (Fig. 5C, D). Based on the Allen Brain Atlas, we set the number of regions to 20 and the number of cell types to 40 for BASS (Additional file 2: Fig. S17). The spatial domain detected from BASS largely resembles that from the Allen Brain Atlas. For example, domains 5 and 9 correspond to the DG, CA3, and CA1 regions of hippocampus while domain 18 corresponds to cortical layer 1. Integrating the identified spatial domains with the identified cell types from BASS, we found that each spatial domain often consists of a unique combination of cell types. For example, the hippocampus consisted of three cell types, with cell type 28 residing in DG, cell type 10 residing in CA3, and cell type 25 residing in CA1. In addition to BASS, we also applied two other cell type clustering methods, Seurat and Garnet, for analysis. For Seurat, we set the resolution parameter to be 0.5 and 1 (Fig. 5E,F and Additional file 2: Fig. S18). Consistent with the results from BASS, Seurat also defined three cell types for hippocampus: cell type 11 residing in DG, cell type 18 residing in CA3, and cell type 15 residing in CA1 [69]. For Garnet, we used the “Brain_Ximerakis_2019” for reference-based cell type inference. With the limitation of gene number, only 17 cell types are mapped in SRT data. Different to the results of BASS and Seurat, Garnet defines only hypendymal cells and neuronal restricted precursors in the hippocampus (Additional file 2: Fig. S19). Based on the results of BASS from the SDD module, we estimated the pseudo-time in the cerebral cortex. The cerebral cortex consists of domains 4, 8, 9, and 10, and we set domain 4 as the starting domain (Fig. 5G). The inferred pseudo-time trajectory is consistent with cortical development, with newer neurons sitting near to the white matter tract [70].

With the detected cell types from each clustering method, the constructed analysis pipeline further performed DEG analysis and identified a total of 2025 and 2159 cell type-specific DEGs (FDR < 0.01) for BASS and Seurat, respectively (Additional file 2: Fig. S20-S21). The identified DEGs are highly overlapped between BASS and Seurat for the same anatomical structure. For example, the number of overlapping DEGs between BASS and Seurat for the DG, CA1, and CA3 is 22 (out of 24 DEGs in cell type 28 for BASS, all the DEGs in cell type 11 for Seurat), 81 (all the DEGs in cell type 10 for BASS, 98 DEGs in cell type 18 for Seurat), and 64 (65 DEGs in cell type 25 for BASS, all the DEGs in cell type 15 for Seurat), respectively.

## Discussion

In summary, we have designed and developed SRT-Server to streamline SRT data analysis and make it more accessible to biological researchers. The advantages of SRT-Server include the following:

1. General data input compatibility. The input format is general and with four different ST platforms, converting them into a consistent file format (H5 file). The h5 format is compatible with both Python and R, accommodating a wide range of analytic software. The format also allows each module to only load the necessary part of the data, thus improving computational efficiency.
2. User-friendly interface along with a comprehensive set of analytic methods, allowing for building user-desired analytic pipelines using various method combinations. SRT-Server offers 13 computational methods implemented through ten analytic modules and provides 51 accompanying datasets to enable comprehensive analysis. Building upon a series of flexible frameworks and a compatible server design, SRT-Server makes building SRT data analysis pipelines akin to “playing LEGO.”
3. Ease of visualization. SRT-Server provides ample visualizations for each procedure and result files, ensuring users have a clear understanding of their data.
4. An extendable framework, which allows computational biologists to integrate their own methods into SRT-Server in the future to make them readily available to biological researchers.
5. A secure user management system that ensures data safety.

Importantly, the implementation of SRT-Server is different from many existing platforms such as Squidpy, Giotto, SPATA2, and Seurat, in four important aspects [11, 13, 14, 23]. First, SRT-Server uses a Lego-like interface for building analytic pipelines for spatial transcriptomics analysis, thus allowing users without any prior coding experience to analyze spatial transcriptomics data and democratizing the analysis of spatial transcriptomics across research domains. Second, SRT-Server is specifically designed for spatial transcriptomics analysis, while existing platforms are either primarily focused on single-cell analysis or contain many tools that are not specifically designed for spatial transcriptomics. For example, the primary dimensional reduction procedure implemented in all existing platforms is principal component analysis (PCA), which works well for single-cell data but does not accommodate spatial correlation information that is necessary for various spatial transcriptomics analytic tasks. In contrast, SRT-Server uses SRT-specific dimension reduction method SpatialPCA [27], which accommodates spatial correlation across measured locations on the tissue and thus facilitates SRT-specific downstream analysis. Third, SRT-Server provides a comprehensive set of advanced and cutting-edge tools for users to choose from in each SRT analytic task. With a total of ten analytic modules and 16 implemented methods, SRT-Server represents the most comprehensive SRT analytic platform available to date. In contrast, the tools for each task in existing SRT platforms are often limited and simple. For example, for deconvolution analysis, Squidpy, SPATA2, and Seurat do not provide any deconvolution tools. Giotto provides only one deconvolution tool, SpatialDeconv, which does not perform well in recent benchmarking studies [18]. While SRT-Server implements three spatial transcriptomics-specific deconvolution methods, including CARD, cell2location and Tangram, which are among the best performing methods in recent benchmarking studies. Finally, SRT-Server provided a wide selection of datasets for deconvolution, cell typing, and cell–cell communication analysis, while most existing platforms do not provide any datasets. For example, for deconvolution analysis, all existing platforms require users to input an annotated scRNA-seq data as a reference panel. In contrast, SRT-Server not only allows users to provide their own reference data, but also, in the absence of such user-provided reference data, offers users the option to perform deconvolution using any of the 51 provided scRNA-seq reference datasets. As another example, SRT-Server provides two cell type annotation methods with 44 reference data sets for annotating the detected cell types in the cell tying analysis, while none of the other platforms offer any of these features. As a third example, for CCC, SRT-Server provides 20 ligand-receptor databases, while Giotto only provides two databases and none of the other platforms provide any databases.

One important future direction of SRT-Server is to extend the server to handle cohort-scale spatial transcriptomics data that will likely appear in the coming years, with diverse experimental groups and specific spatial features. Cohort-scale spatial transcriptomics data introduces important statistical and computational challenges that are not yet well addressed in the field, as there is currently limited data availability for cohort-scale spatial transcriptomics. Specifically, the largest spatial transcriptomics data to date contains only 12 samples [71, 72]. Because of a lack of large cohort data, almost all existing spatial transcriptomics studies have focused on analyzing one tissue slice at a time, and only a few notable exceptions of computational tools can handle multiple tissue slices or multiple tissue samples. For example, the BASS method incorporated in our SDD module on the SRT-Server can integrate SRT data from multiple tissue samples for detecting cell type clusters and spatial domains [22]. However, while it remains a challenge to analyze cohort-scale spatial transcriptomics data, we implemented multiple additional features to SRT-Server to make it ready for analyzing the potentially large cohort of spatial transcriptomics data as they will likely emerge in the next few years. First, we have modified the data input framework, adding a drop-down manual in the *Input Data*, so that multiple datasets can be uploaded to SRT-Server and jointly analyzed there. Second, we have implemented in SRT-Server multiple data integration methods developed for scRNA-seq data analysis, including *merge*, *harmony*, and *integration* in Seurat, to be used for spatial transcriptomics integration across multiple datasets. In particular, all three methods in Seurat are incorporated in the CT module and the *merge* and *harmony* functions in Seurat are also incorporated in the SDD module as options for data integration before running BASS. Finally, we have also implemented BASS in the SDD module, which can directly model multiple tissue slices in spatial transcriptomics. Importantly, we applied the newly added options in the real-data applications for analyzing multiple tissues slices collected from different individuals. In particular, we applied two analytic modes of BASS to jointly analyze two tissue slices for spatial domain detection in Case Study 2. The two modes of BASS include a batch corrected mode where Harmony was used to correct for batch effects before analysis and a batch uncorrected mode where BASS is directly applied to the data without batch effect correction (Additional File 2: Fig. 22). The results highlight the importance of performing batch correction before joint tissue slice analysis, supporting the benefits of SRT-Server in analyzing large spatial transcriptomics.

## Conclusions

Overall, SRT-Server presents a user-friendly, efficient, effective, secure, and expandable solution for SRT data analysis, opening new doors for researchers in the field. In addition, while SRT-Server is specifically designed for SRT data analysis, its architecture lays the groundwork for the development of additional computational web servers that cater to a wide variety of omics data analysis needs.

## Availability and requirements

Project name: SRT-Server.

Project home page: https://github.com/biostat0903/SRT-Server, https://spatialtranscriptomicsanalysis.com/.

Operating systems: Windows, macOS, Linux.

Programming language: R, python, JavaScript, HTML.

License: MIT.

### Supplementary Information

**Additional file 1:** **Supplementary Tables. Table S1.** Summary for the scRNA-seq data with cell type annotation for different tissue in human and mouse. **Table S2.** Summary for the maker information for different tissue in human. **Table S3.** Computational consumption for three case studies. **Table S4**. Summary for association between genes associated with EMT and pseudo-time.

**Additional file 2: **Differences between SRT-Server and the two analytic tools from 10x Genomics.

**Additional file 3: Supplementary Figures. **Pipelines of three cases analyzed in SRT-Server and additional figures generated from SRT-Server to support the three case studies.** Fig. S1.** Pipeline of case study 1 in SRT-Server. We used QC, DECON, DECON_PY, TRAJ, SDD, DEG, ORA, and CCC modules with its corresponding plot module. **Fig. S2.** Summary for the reference scRNA-seq of mouse brain data. A) Cell type compositions of the reference scRNA-seq data. Each sample includes 20 cell type composition: Oligodendrocytes, Oligodendrocyte precursor cells, Olfactory ensheathing glia, mature Neurons, Neuroendocrine cells, immature Neurons, Arachnoid barrier cells, Vascular and leptomeningeal cells, Endothelial cells, Vascular smooth muscle cells, Astrocytes, Neural stem cells, Astrocyte restricted precursors, Neuronal restricted precursors, Pericytes, Hemoglobin expressing vascular cells, Choroid plexus epithelial cells, Tanycytes, Ependymocytes, Hypendymal cells. B) The heatmap of the top five marker genes for each cell type. **Fig. S3.** Scatter plot of cell type proportion distributions across spatial locations in the mouse brain sagittal anterior. Specifically, cell type proportions are estimated by CARD using the reference consisting of 20 cell types. Here, for each cell type, the cell type proportion is scaled to 0-1 range. Color is showed to represent the 0-1 range of cell type proportions correspondingly. **Fig. S4.** Scatter plot of cell type proportion distribution in the refined spatial map for the mouse brain sagittal anterior. CARD provides an additional analysis to show the cell type proportion in an enhanced grided spatial locations. Here, the resolution is set to be 2,000. **Fig. S5.** Scatter plots display the correlation between three deconvolution methods in Case Study 1. *x*-axis shows the cell type proportion estimated by cell2location and tangram. *y*-axis shows the cell type proportion estimated by CARD. The corresponding relationship between cell type and color is consistent with the pie plot of DECON. **Fig. S6.** Results summary for the SDD module with SpatialPCA for the mouse brain sagittal anterior. A) The location plot with domain number to 10. B) The location plot with domain number to 20. C) The feature plot for the top ten HVGs. **Fig. S7.** The heatmap of the 15 domains from SpatialPCA with top five DEGs. We use SpatialPCA to cluster the spots into 15 domains. The heatmap shows the top five DEGs for each domain. **Fig. S8.** Pipeline of case study 2 in SRT-Server. We used QC, DECON, SDD, DEG, ORA, CCC, and TRAJ module with its corresponding plot module. **Fig. S9.** Summary for the reference scRNA-seq of BRCA HER2+ data. A) Cell type compositions of the reference scRNA-seq data. Each sample includes 17 cell type composition: Endothelial, CAFs, PVL, B cells Memory, B cells Naive, CD8_T_cells, CD4_T_cells, NK cells, Cycling T-cells, NKT cells, Macrophage, Monocyte, Cycling Myeloid, DCs, Normal Epithelial, Plasmablasts, Cancer Epithelial. B) The heatmap of marker gene for each cell type. B) The heatmap of the top five marker genes for each cell type. **Fig. S10.** Scatter plot of cell type proportion distributions across spatial locations in the BRCA HER2+ samples. Specifically, cell type proportions are estimated by CARD using the reference consisting of 17 cell types. Here, for each cell type, the cell type proportion is scaled to 0-1 range. Color is showed to represent the 0-1 range of cell type proportions correspondingly. **Fig. S11.** Results summary for the SDD module with SpatialPCA for the BRCA HER2+ samples. A) The location plot with domain number to 10. B) The location plot with domain number to 20. C) The feature plot for the top ten HVGs. **Fig. S12.** Summary for TRAJ analysis for BRCA HER2+ samples. A) The relationship between pseudo-time and the gene expression. The *x* and *y *axis shows the pseudo-time and gene expression, respectively. B) Heatmap for gene expression and pseudo-time. **Fig. S13.** The heatmap of the 10 domains from SpatialPCA with top five DEGs for BRCA HER2+ samples. We use SpatialPCA to cluster the spots into 10 domains. The heatmap shows the top five DEGs for each domain. **Fig. S14.** Bubble plot from ORA analysis. Bubble plot displays –log10(p-values) for different pathways from the ORA module (*y*-axis). Pathways are colored by seven categories: DO (red), GO biological process (yellow), GO cellular component (sky blue), GO molecular function (green), KEGG (chocolate2), Reactome (blue), and Wikipathways (grass-green). **Fig. S15.** The circle plot for each cell type for BRCA HER2+. Each plot shows the number of significant ligand-receptor pairs from a specific cell type to other cell types. The edge width is proportional to the indicated number of ligand-receptor pairs. **Fig. S16.** Pipeline of case study 3 in SRT-server. We used QC, CT, DEG, and TRAJ module with its corresponding plot module. Note that we added three CT modules with different methods, which results in the output of DEG are different. TRAJ module is only followed by CT with BASS. **Fig. S17.** Summary for the CL_jo module with BASS. A) The location plot for spatial domain with domain number = 20 and cell type number = 35. B) The location plot for spatial domain with domain number = 25 and cell type number = 35. C) The location plot for spatial domain with domain number = 25 and cell type number = 40. D) The location plot for cell type with domain number = 20 and cell type number = 35. E) The location plot for cell type with domain number = 25 and cell type number = 35. F) The location plot for cell type with domain number = 25 and cell type number = 40. **Fig. S18.** Summary for the clustering result from Seurat. A) The location plot of cell type with resolution = 0.5. B) The UMAP plot with resolution = 0.5. **Fig. S19. **17. cell types annotated by Garnett in the CL_annot module. **Fig. S20.** The Heatmap for BASS with top five DEGs. We use BASS to cluster the cells into 33 domains. The heatmap shows the top five DEGs for each domain. **Fig. S21.** The Heatmap from Seurat (resolution = 1) with top five DEGs. We use Seurat to cluster the cells into 30 cell types. The heatmap shows the top five DEGs for each domain. **Fig. S22.** Location plots display the domain clustering results from BASS on different samples with or without the data integration step. The number of spatial domains is set to be 10 and the number of cell types is set to be 20. A) BASS results after a data integration step with Harmony; B) BASS results without data integration.

## Data Availability

This study made use of multiple publicly available datasets. For Case Study 1, the data was obtained from the 10X Visium spatial gene-expression repository (https://www.10xgenomics.com/resources/datasets/mouse-brain-serial-section-2-sagittal-anterior-1-standard) [5]. A raw count matrix was kindly shared by Dr. Methodios Ximerakis for the DECON and DECON_py module while a normalized gene expression matrix was obtained in GSE129788 (https://www.ncbi.nlm.nih.gov/geo/query/acc.cgi?acc=GSE129788) for the analysis of other modules [50]. For Case Study 2, the SRT data in 10X Visium format was kindly shared by Dr. Tong Liu and Prof. Hongquan Zhang for the QC module while the SRT expression matrix was obtained in GSE190811 (https://www.ncbi.nlm.nih.gov/geo/query/acc.cgi?acc=GSE190811) [10]. The corresponding reference scRNA-seq was obtained from GSE176078 (https://www.ncbi.nlm.nih.gov/geo/query/acc.cgi?acc=GSE176078) [60]. For Case Study 3, the data (slice 2 replication 3) was obtained from (https://info.vizgen.com/mouse-brain-data) and a quarter of the slice centering on hippocampus was selected for analysis (https://1drv.ms/f/s!ApfEV21lbccRg7te9Jq6HTJb28DaxA?e=dcD7GU) [8]. Databases used in the present study include Allen Brain Atlas (https://www.brain-map.org) [49]. Source data are provided with this paper. The back-end R code of SRT-Server is publicly available at https://github.com/biostat0903/SRT-Server. The source code is released under the GNU General Public License version 3 (GPL >  = 3). The website of SRT-Server is free to use at https://spatialtranscriptomicsanalysis.com/.

## Abbreviations

SRT: Spatially resolved transcriptomics
SVGs: Spatially variable genes
DEGs: Differentially expressed genes
scRNA-seq: Single-cell RNA-seq
APIs: Application programming interfaces
SPA: Single page application
SSL: Secure socket layer
GUI: Graphical user interface
QC: Quality control
miRNA: MicroRNA
lncRNA: Long non-coding RNA
GLSM: Generalized linear spatial model
PQL: Penalized quasi-likelihood
DECON: Deconvolution
CAR: Conditional autoregressive
SDD: Spatial domains detection
SpatialPCA: Spatially aware principal component analysis
BASS: Bayesian analytics for spatial segmentation
CT: Cell typing
PCA: Principal component analysis
CCA: Canonical correlation analysis
UMAP: The Uniform Manifold Approximation and Projection
ORA: Over representative analysis
GO: Gene Ontology
KEGG: Kyoto Encyclopedia of Genes and Genomes
DO: Disease Ontology
CCC: Cell Cell Communication
LR: Ligand-receptor
KNN: K Nearest Neighborhood
TRAJ: Pseudo-time trajectory
H&E: Hematoxylin and eosin
OB: Olfactory bulb
LNM: Lymph node metastasis
CAF: Cancer-associated fibroblasts
iCAFs: Inflammatory-like CAFs
