# Supplementary material for: SRT-Server: powering the analysis of spatial transcriptomic data
Source: Genome Med. 2024 Jan 26;16:18. doi: 10.1186/s13073-024-01288-6 (PMC10811909; doi:10.1186/s13073-024-01288-6)
Supplement: Supplementary file 2 — Additional file 2: Differences between SRT-Server and the two analytic tools from 10x Genomics. [file 13073_2024_1288_MOESM2_ESM.docx]

**Differences between SRT-Server and the two analytic tools from 10x Genomics**

10x Genomics provides two analytic tools for SRT data analysis: i) Space Ranger (v.2.0, the last version), which performs alignment, detects tissue barcodes, and carries out secondary analysis of gene expression; ii) Loupe Browser (v.7.0.0, the last version), which can be used to visualize output from Space Ranger in “cloupe” format. The analysis pipeline in Space Ranger contains a fixed and relatively limited set of secondary analysis tools for gene expression and these include clustering analysis, t-SNE and UMAP projections as well as differential gene expression analysis between clusters.

Compared to these tools, SRT-Server is primarily focused on downstream analysis while Space Ranger/Loupe Browser are primarily focused on upstream analysis (Fig. 1). As a result, SRT-Server provides a much more comprehensive set of SRT specific analytic methods that can be widely applied for various SRT downstream analytic tasks. The unique analytic tasks that can be carried out by SRT-Server but not Space Ranger/Loupe Browser include cell-cell communication, SVG identification, spatial domain detection, gene set enrichment analysis, or pseudo-time trajectory analysis. Even for the downstream analytic tasks that can be carried out by both SRT-Server and Space Ranger/Loupe Browser, SRT-Server provides a much more comprehensive selection of analytic methods. For example, for cell type deconvolution in spatial transcriptomics, SRT-Server provides three mainstream SRT deconvolution methods, including CARD (both reference-based and reference-free), cell2location and Tangram, with 51 reference single cell RNA-seq panels available for users to choose from. In contrast, Space Ranger only provides a single reference-free method, STdeconvolve, which has relatively poor performance in recent benchmarking studies [1, 2]. As another example, for cell type clustering in single-cell resolution SRT data, SRT-Server provided two methods, including both an accurate SRT specific method BASS and an efficient generic method PCA as implemented in Seurat. In contrast, Space Ranger only provides the option of PCA which is not as accurate as BASS for many SRT datasets. As a third example, for DE analysis, SRT-Server implements the Wilcox test which is known to be much more robust and efficient for DE analysis compared to DESeq2 provided by Space Ranger [3]. As a fourth example, SRT-Server provides the integration setting for SDD/CT methods that allows for the joint analysis of multiple tissue slices. In contrast, Space Ranger/Loupe Browser only supports single tissue slice analysis.

In addition, SRT-Server provides various plotting and visualization features including some of the specific interactive features that 10x Genomics tools are not well equipped to handle. For example, the interactive features in SRT-Server, such as those implemented in SDD_Plot/CT_Plot and CCC_Plot, can be performed on multiple tissue slices, while the interactive operation in Loupe Brower can only be applied to one tissue slice. As another example, SRT-Server provides bubble plot and feature plot for DEGs, while such plots are not included in Loupe Brower. As a third example, SRT-Server provides a range of location plots across different parameter settings in the SDD/CT module, so that the user can easily determine the optimal parameter setting such as the number of spatial domains set in SpatialPCA. Loupe Browser instead can only visualize a location plot for one parameter at a time.

**Reference**

1. Miller BF, Huang F, Atta L, Sahoo A, Fan J: **Reference-free cell type deconvolution of multi-cellular pixel-resolution spatially resolved transcriptomics data.** *Nature Communications* 2022, **13:**2339.

2. Li H, Zhou J, Li Z, Chen S, Liao X, Zhang B, Zhang R, Wang Y, Sun S, Gao X: **A comprehensive benchmarking with practical guidelines for cellular deconvolution of spatial transcriptomics.** *Nature Communications* 2023, **14:**1548.

3. Li Y, Ge X, Peng F, Li W, Li JJ: **Exaggerated false positives by popular differential expression methods when analyzing human population samples.** *Genome Biology* 2022, **23:**79.
