# Supplementary material for: SRT-Server: powering the analysis of spatial transcriptomic data
Source: Genome Med. 2024 Jan 26;16:18. doi: 10.1186/s13073-024-01288-6 (PMC10811909; doi:10.1186/s13073-024-01288-6)
Supplement: Supplementary file 3 — Additional file 3: Supplementary Figures. Pipelines of three cases analyzed in SRT-Server and additional figures generated from SRT-Server to support the three case studies. Fig. S1. Pipeline of case study 1 in SRT-Server. We used QC, DECON, DECON_PY, TRAJ, SDD, DEG, ORA, and CCC modules with its corresponding plot module. Fig. S2. Summary for the reference scRNA-seq of mouse brain data. A) Cell type compositions of the reference scRNA-seq data. Each sample includes 20 cell type composition: Oligodendrocytes, Oligodendrocyte precursor cells, Olfactory ensheathing glia, mature Neurons, Neuroendocrine cells, immature Neurons, Arachnoid barrier cells, Vascular and leptomeningeal cells, Endothelial cells, Vascular smooth muscle cells, Astrocytes, Neural stem cells, Astrocyte restricted precursors, Neuronal restricted precursors, Pericytes, Hemoglobin expressing vascular cells, Choroid plexus epithelial cells, Tanycytes, Ependymocytes, Hypendymal cells. B) The heatmap of the top five marker genes for each cell type. Fig. S3. Scatter plot of cell type proportion distributions across spatial locations in the mouse brain sagittal anterior. Specifically, cell type proportions are estimated by CARD using the reference consisting of 20 cell types. Here, for each cell type, the cell type proportion is scaled to 0-1 range. Color is showed to represent the 0-1 range of cell type proportions correspondingly. Fig. S4. Scatter plot of cell type proportion distribution in the refined spatial map for the mouse brain sagittal anterior. CARD provides an additional analysis to show the cell type proportion in an enhanced grided spatial locations. Here, the resolution is set to be 2,000. Fig. S5. Scatter plots display the correlation between three deconvolution methods in Case Study 1. x-axis shows the cell type proportion estimated by cell2location and tangram. y-axis shows the cell type proportion estimated by CARD. The corresponding relationship between cell type and color is consisten [file 13073_2024_1288_MOESM3_ESM.docx]

**Supplementary Figures**


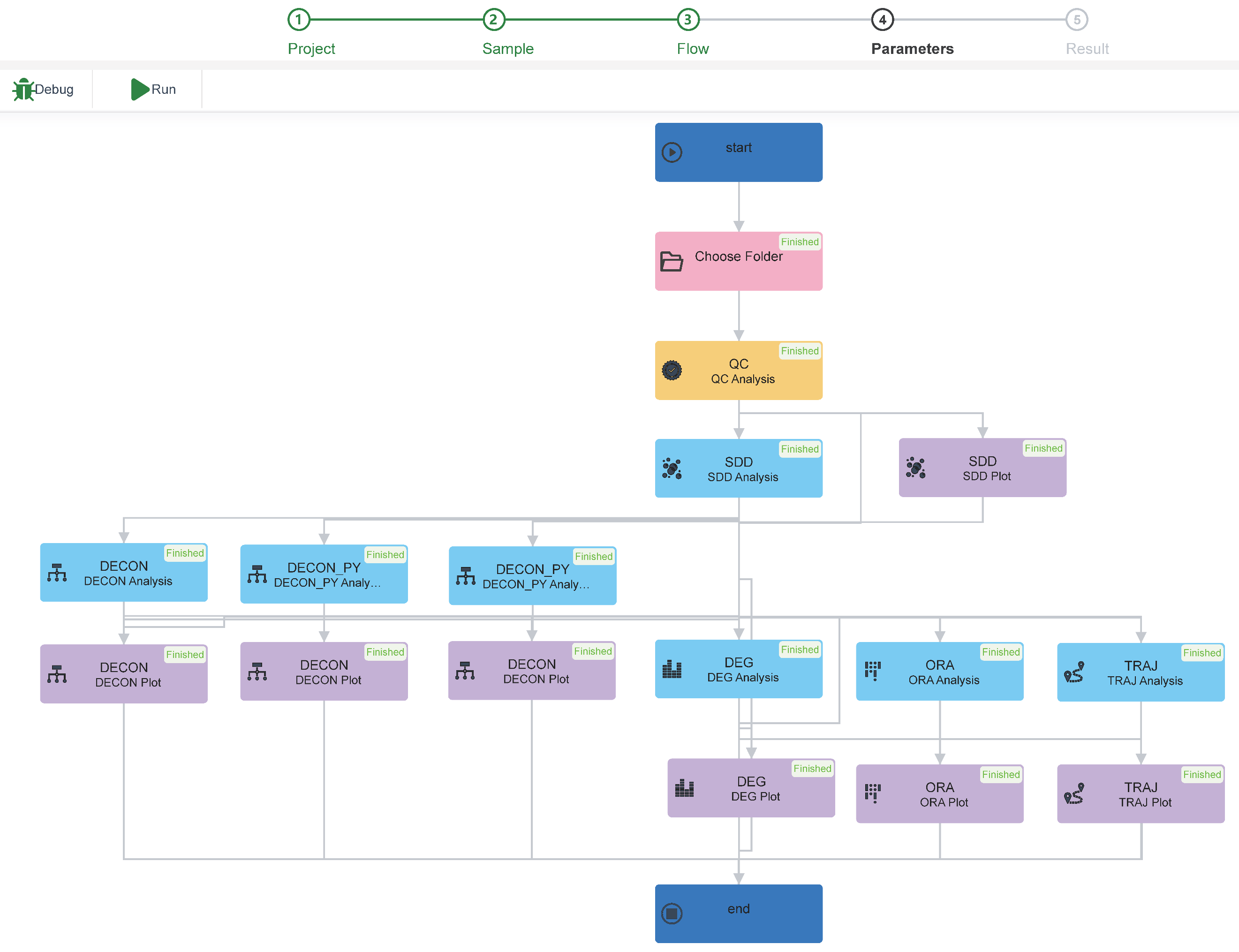


**Fig. S1 Pipeline of case study 1 in SRT-Server.** We used QC, DECON, DECON_PY, TRAJ, SDD, DEG, ORA, and CCC modules with its corresponding plot module.

**
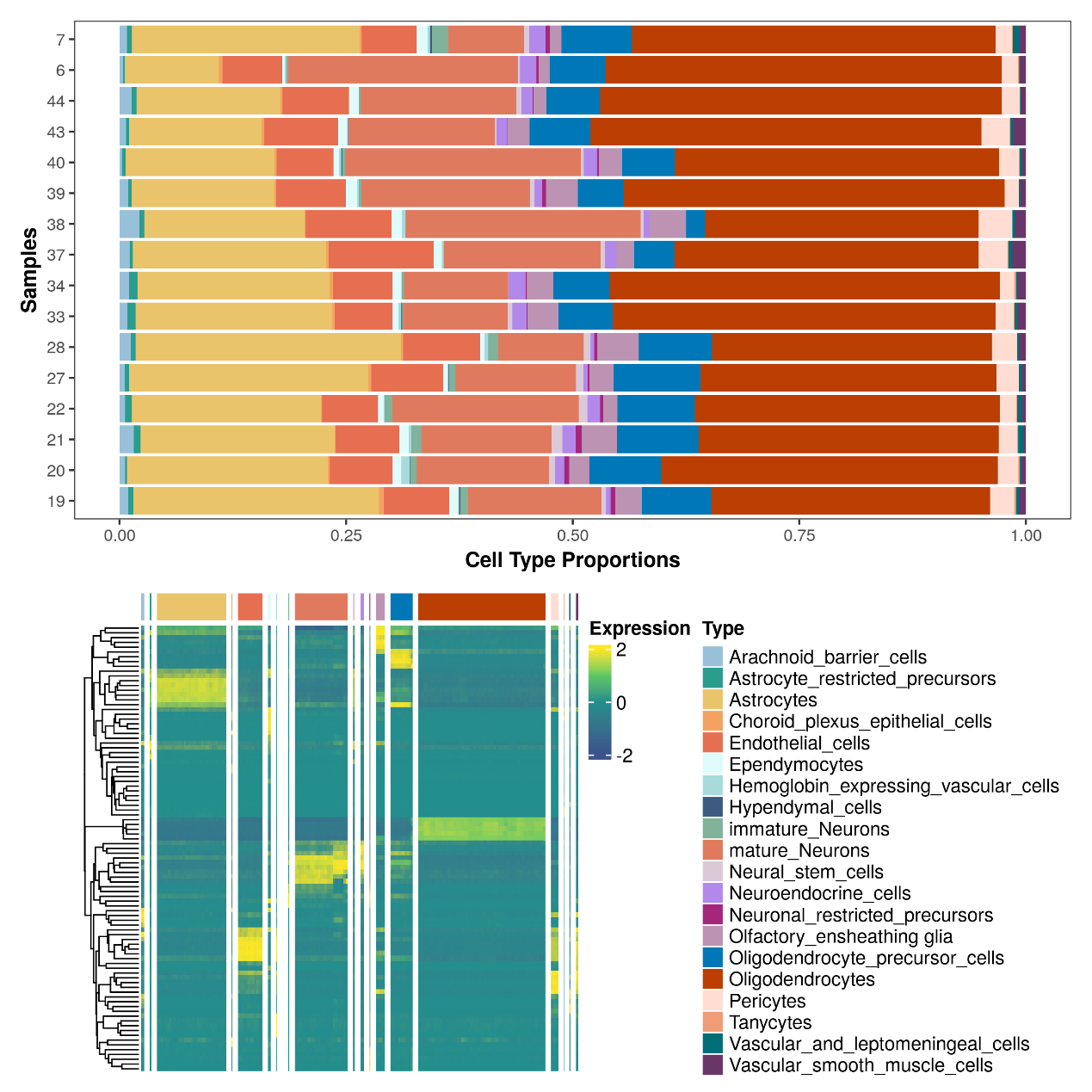
**

**Fig. S2 Summary for the reference scRNA-seq of mouse brain data.** A) Cell type compositions of the reference scRNA-seq data. Each sample includes 20 cell type composition: Oligodendrocytes, Oligodendrocyte precursor cells, Olfactory ensheathing glia, mature Neurons, Neuroendocrine cells, immature Neurons, Arachnoid barrier cells, Vascular and leptomeningeal cells, Endothelial cells, Vascular smooth muscle cells, Astrocytes, Neural stem cells, Astrocyte restricted precursors, Neuronal restricted precursors, Pericytes, Hemoglobin expressing vascular cells, Choroid plexus epithelial cells, Tanycytes, Ependymocytes, Hypendymal cells. B) The heatmap of the top five marker genes for each cell type.


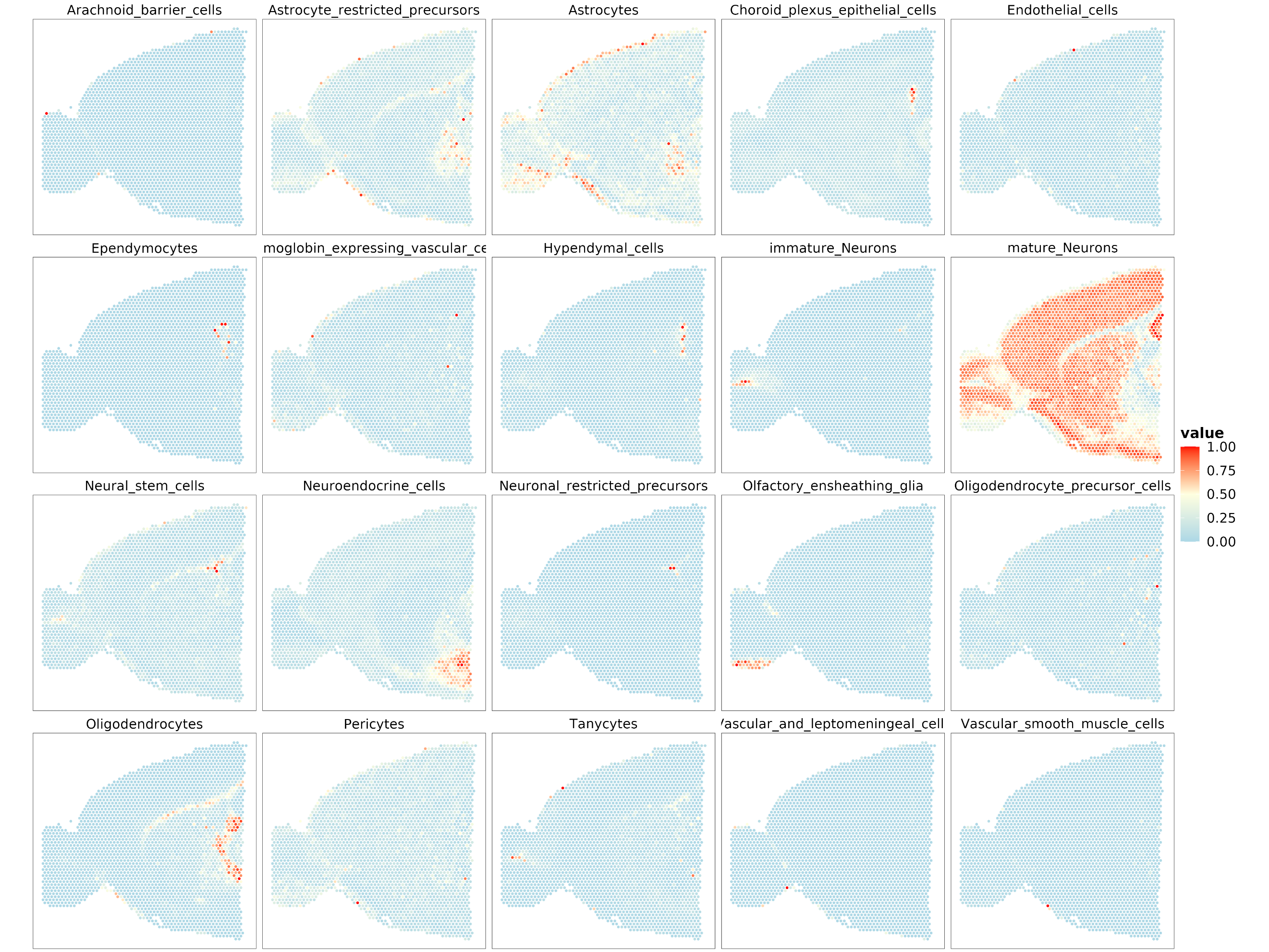


**Fig. S3 Scatter plot of cell type proportion distributions across spatial locations in the mouse brain sagittal anterior.** Specifically, cell type proportions are estimated by CARD using the reference consisting of 20 cell types. Here, for each cell type, the cell type proportion is scaled to 0-1 range. Color is showed to represent the 0-1 range of cell type proportions correspondingly.


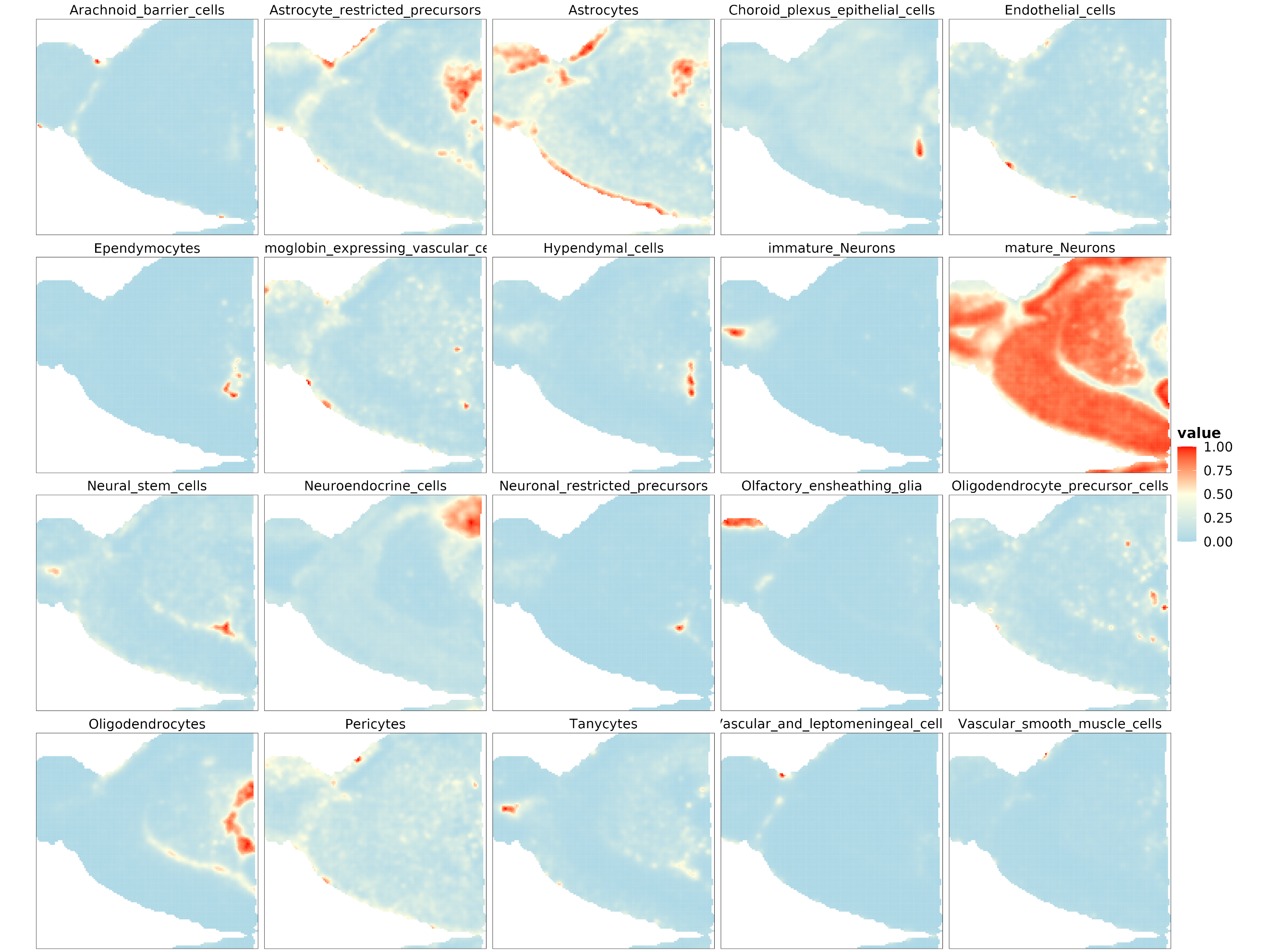


**Fig. S4 Scatter plot of cell type proportion distribution in the refined spatial map for the mouse brain sagittal anterior.** CARD provides an additional analysis to show the cell type proportion in an enhanced resolution. Each point is the imputed cell type proportion and corresponding newly grided spatial locations. Here, the resolution is set to be 2,000.

**
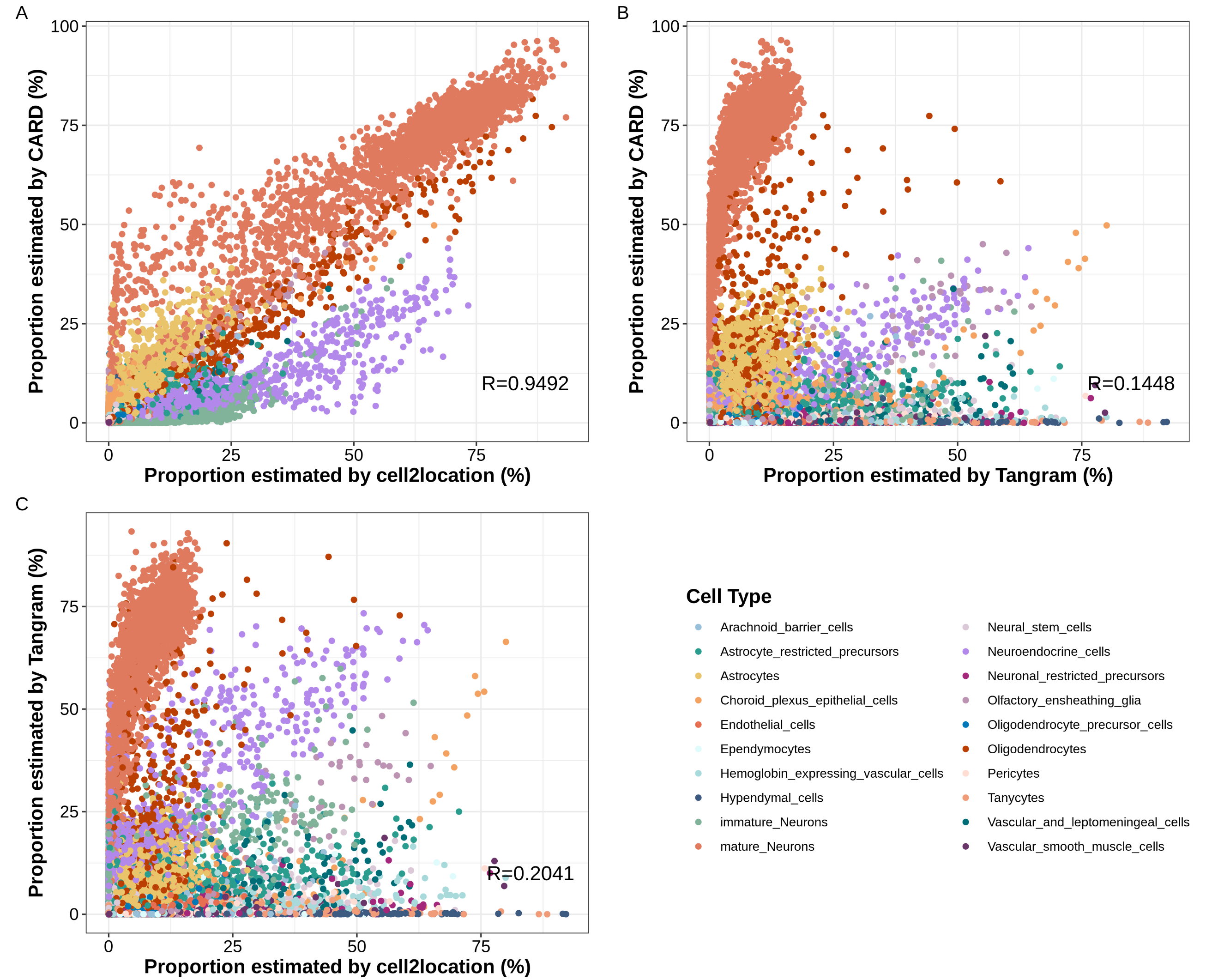
**

**Fig. S5** **Scatter plots display the correlation between three deconvolution methods in Case Study 1.** *x*-axis shows the cell type proportion estimated by cell2location and tangram. *y*-axis shows the cell type proportion estimated by CARD. The corresponding relationship between cell type and color is consistent with the pie plot of DECON.


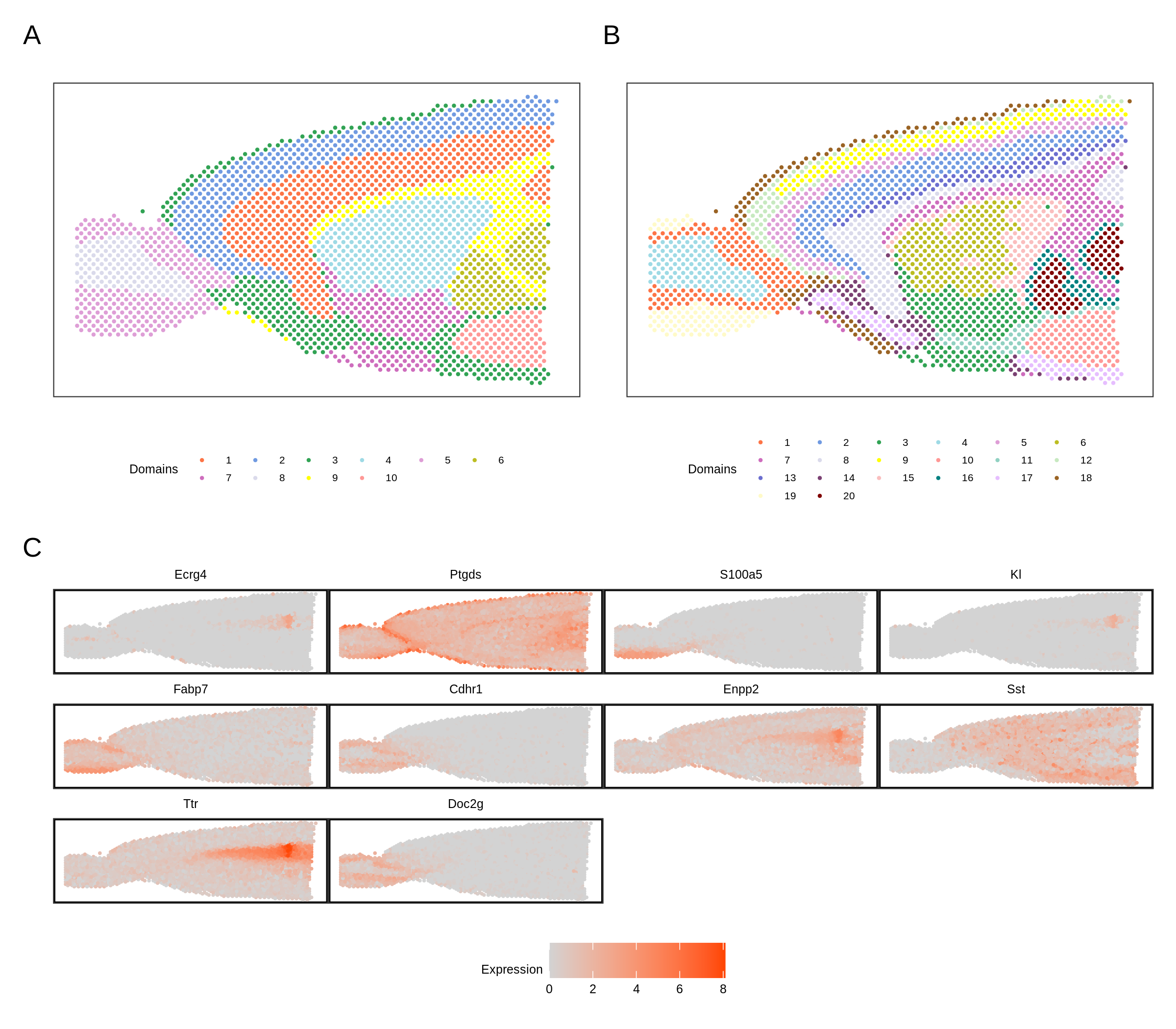


**Fig. S6 Results summary for the SDD module with SpatialPCA for the mouse brain sagittal anterior.** A) The location plot with domain number to 10. B) The location plot with domain number to 20. C) The feature plot for the top ten HVGs.


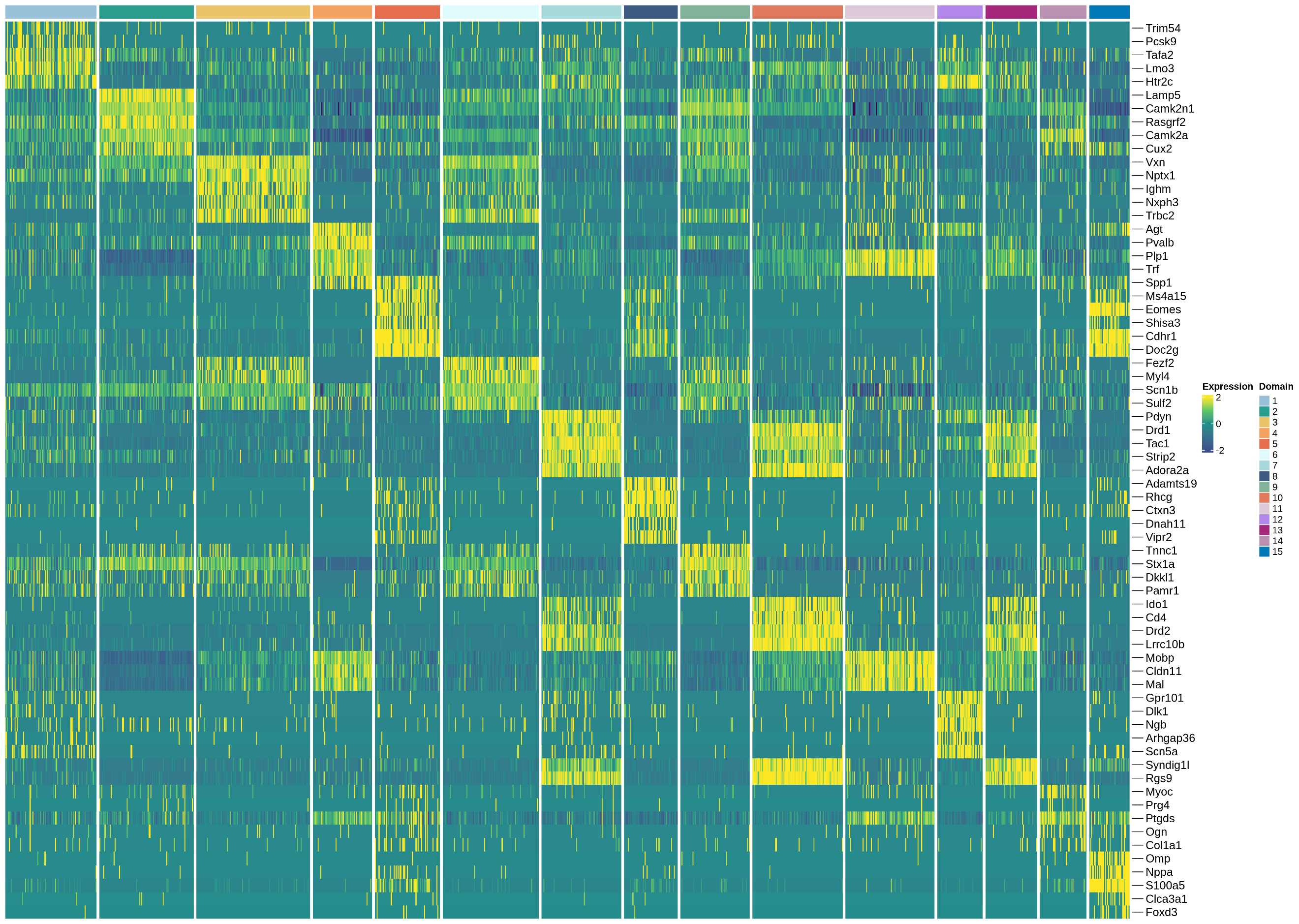


**Fig. S7 The heatmap of the 15 domains from SpatialPCA with top five DEGs.** We use SpatialPCA to cluster the spots into 15 domains. The heatmap shows the top five DEGs for each domain.


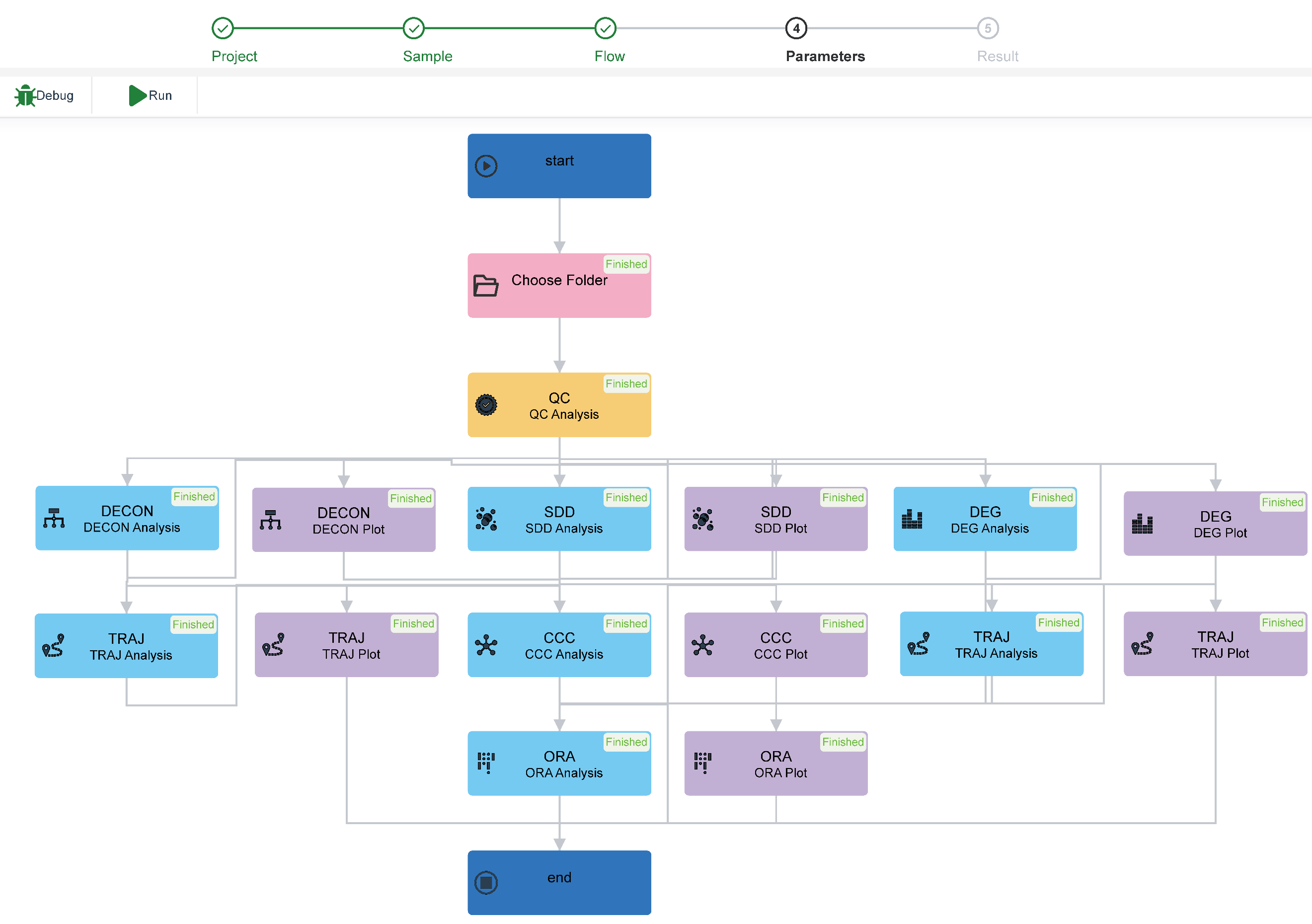


**Fig. S8 Pipeline of case study 2 in SRT-Server.** We used QC, DECON, SDD, DEG, ORA, CCC, and TRAJ module with its corresponding plot module.


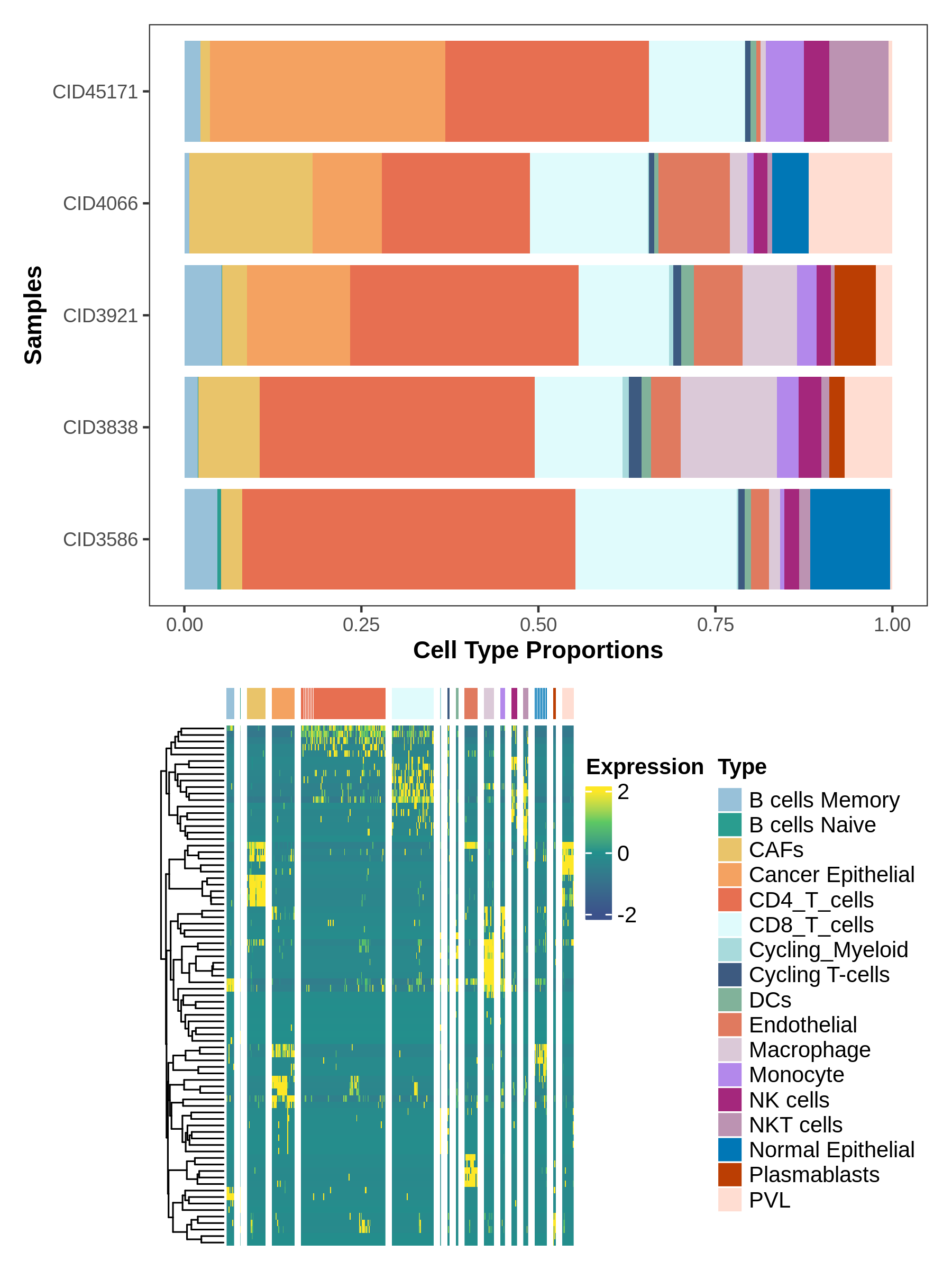


**Fig. S9 Summary for the reference scRNA-seq of BRCA HER2+ data.** A) Cell type compositions of the reference scRNA-seq data. Each sample includes 17 cell type composition: Endothelial, CAFs, PVL, B cells Memory, B cells Naive, CD8_T_cells, CD4_T_cells, NK cells, Cycling T-cells, NKT cells, Macrophage, Monocyte, Cycling Myeloid, DCs, Normal Epithelial, Plasmablasts, Cancer Epithelial. B) The heatmap of marker gene for each cell type. B) The heatmap of the top five marker genes for each cell type.


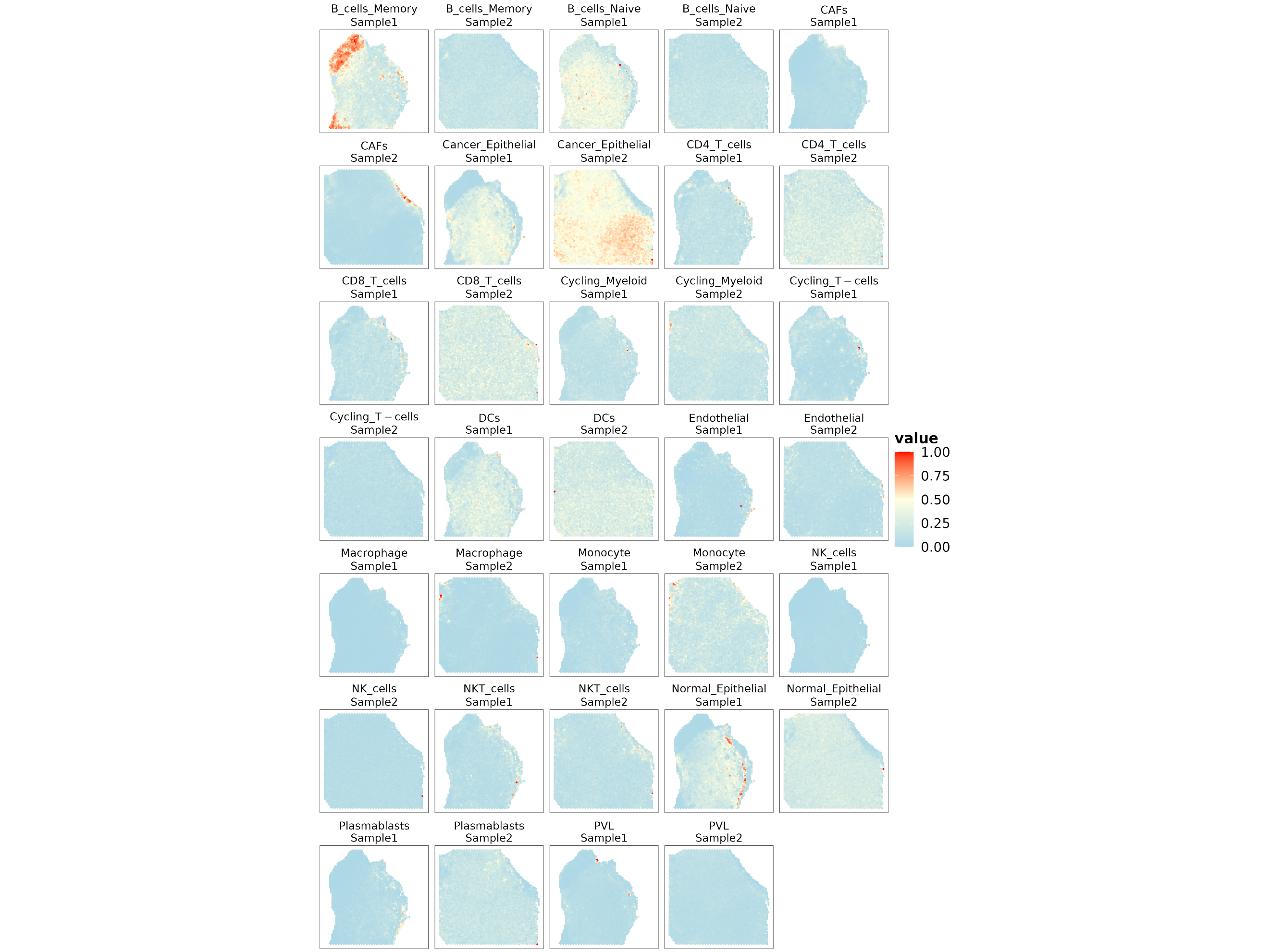


**Fig. S10 Scatter plot of cell type proportion distributions across spatial locations in the BRCA HER2+ samples.** Specifically, cell type proportions are estimated by CARD using the reference consisting of 17 cell types. Here, for each cell type, the cell type proportion is scaled to 0-1 range. Color is showed to represent the 0-1 range of cell type proportions correspondingly.


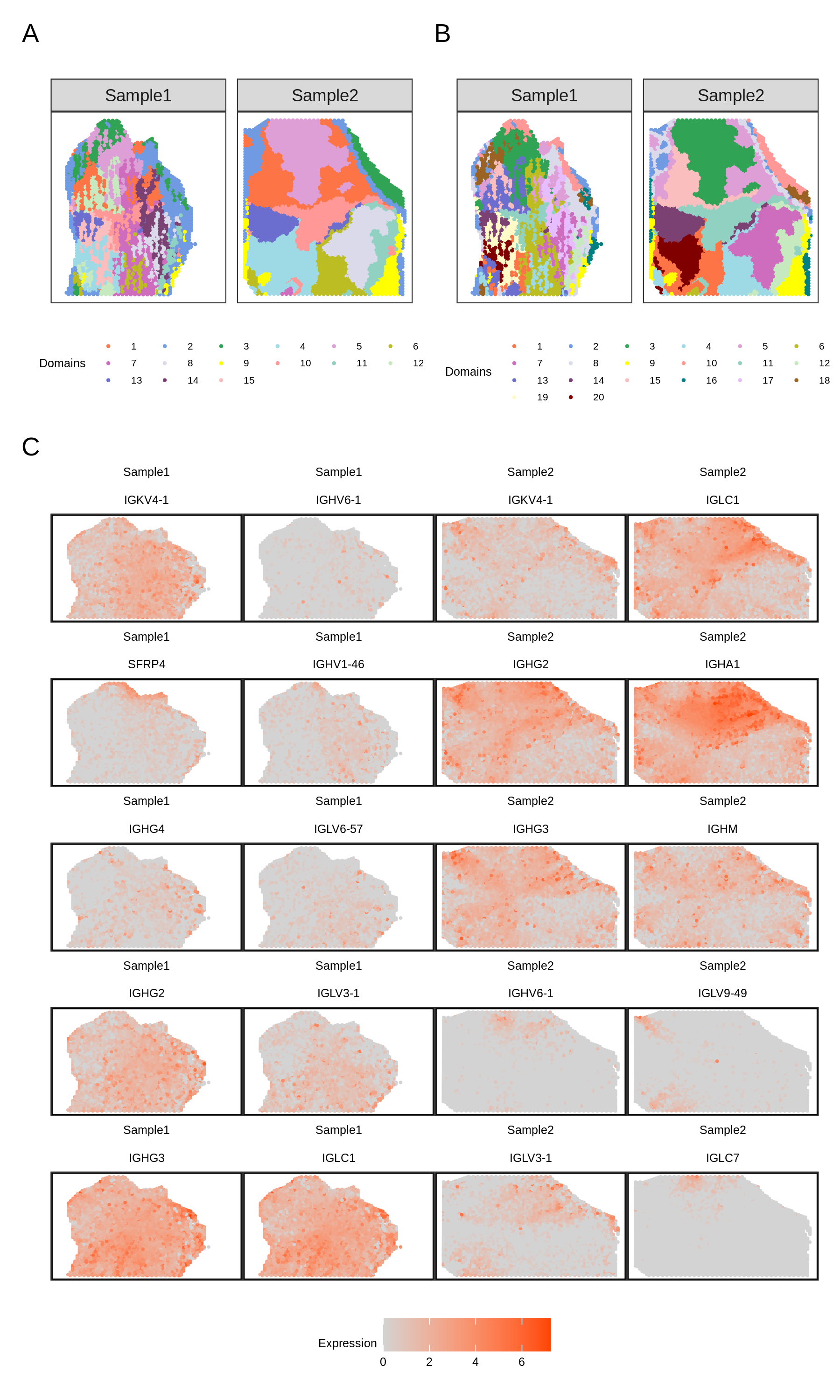


**Fig. S11 Results summary for the SDD module with SpatialPCA for the BRCA HER2+ samples.** A) The location plot with domain number to 10. B) The location plot with domain number to 20. C) The feature plot for the top ten HVGs.


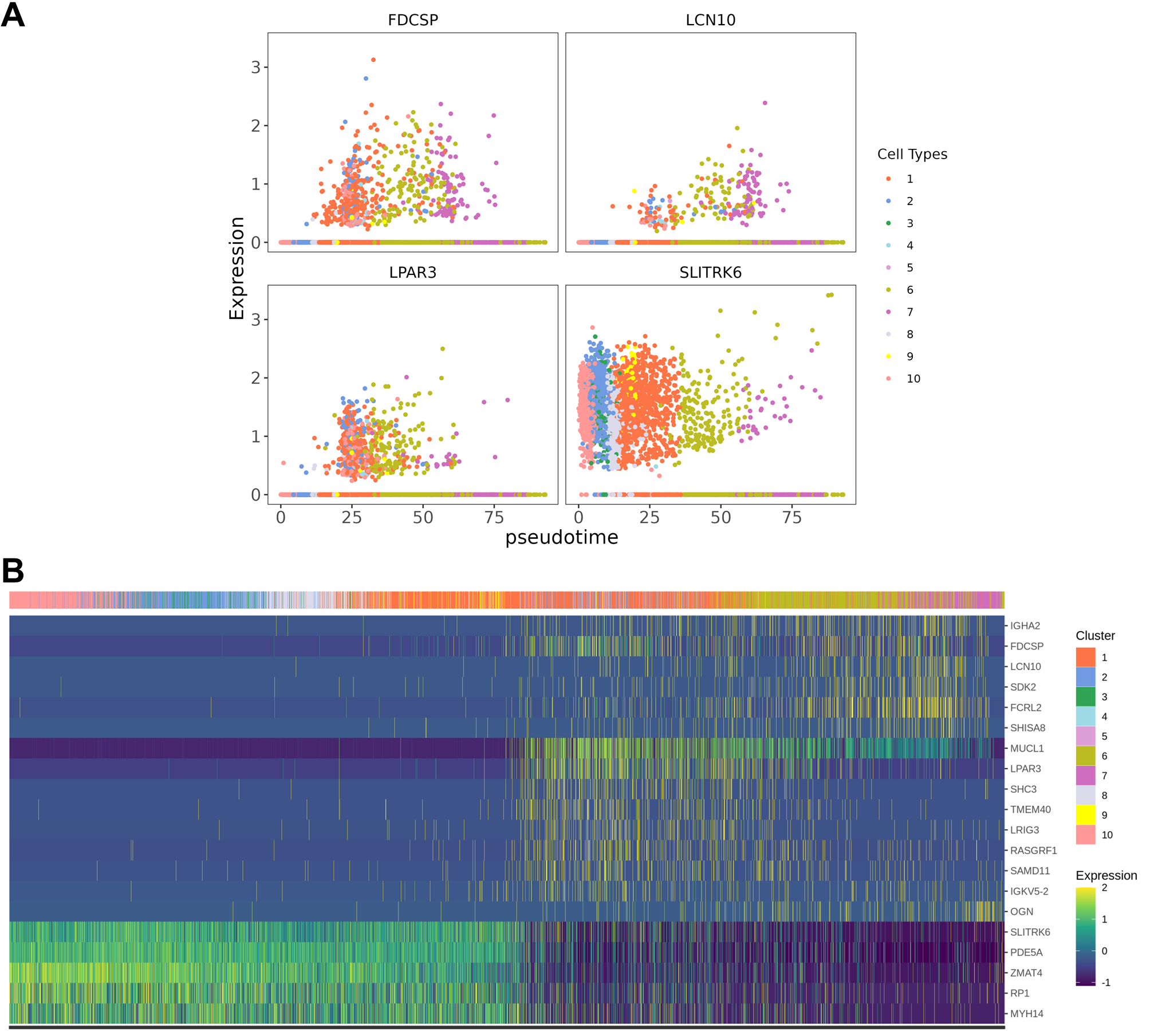


**Fig. S12 Summary for TRAJ analysis for BRCA HER2+ samples.** A) The relationship between pseudo-time and the gene expression. The *x* and *y* axis shows the pseudo-time and gene expression, respectively. B) Heatmap for gene expression and pseudo-time.


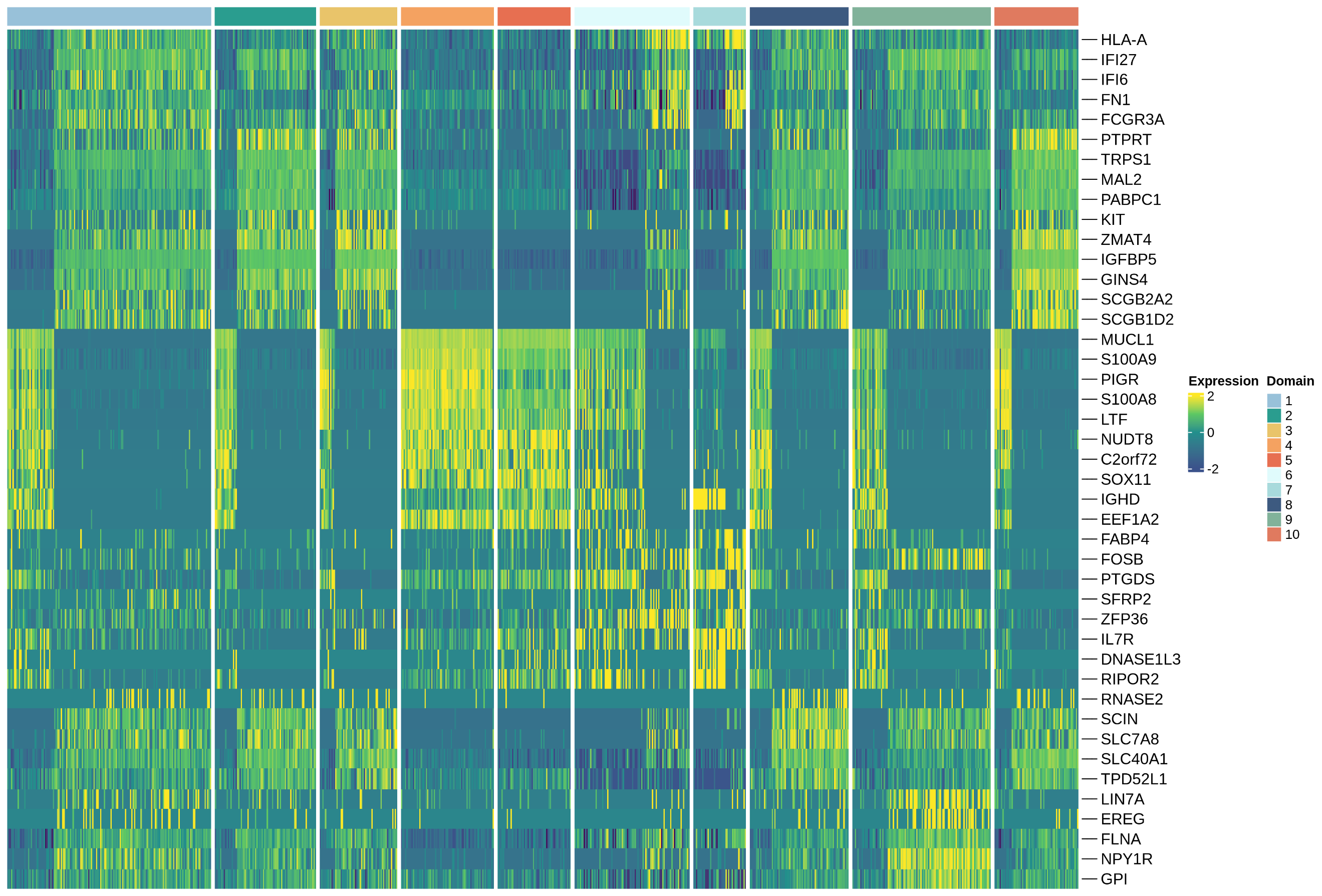


**Fig. S13 The heatmap of the 10 domains from SpatialPCA with top five DEGs for BRCA HER2+ samples.** We use SpatialPCA to cluster the spots into 10 domains. The heatmap shows the top five DEGs for each domain.


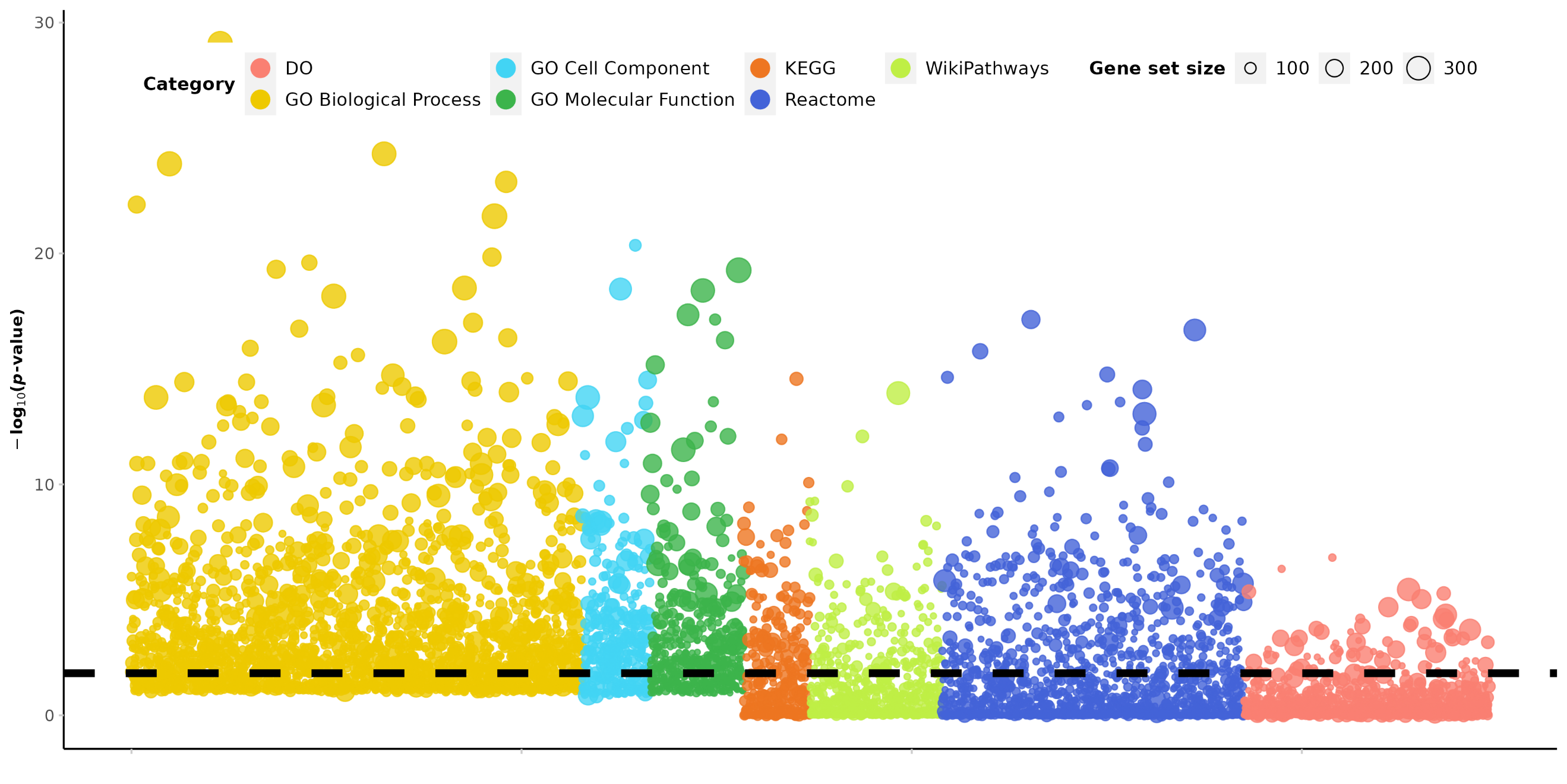


**Fig. S14 Bubble plot from ORA analysis.** Bubble plot displays –log10(p-values) for different pathways from the ORA module (*y*-axis). Pathways are colored by seven categories: DO (red), GO biological process (yellow), GO cellular component (sky blue), GO molecular function (green), KEGG (chocolate2), Reactome (blue), and Wikipathways (grass-green).


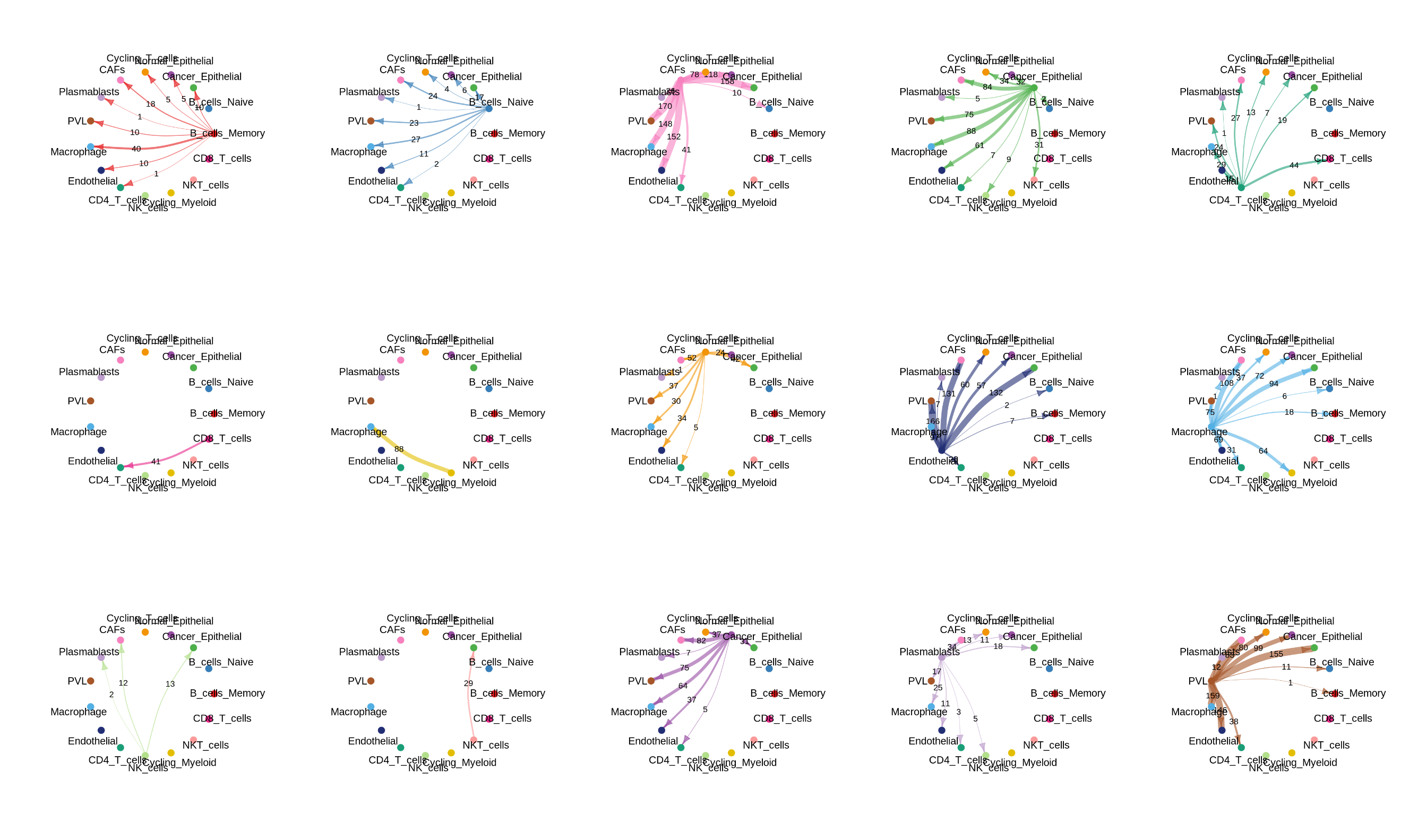


**Fig. S15 The circle plot for each cell type for BRCA HER2+.** Each plot shows the number of significant ligand-receptor pairs from a specific cell type to other cell types. The edge width is proportional to the indicated number of ligand-receptor pairs.


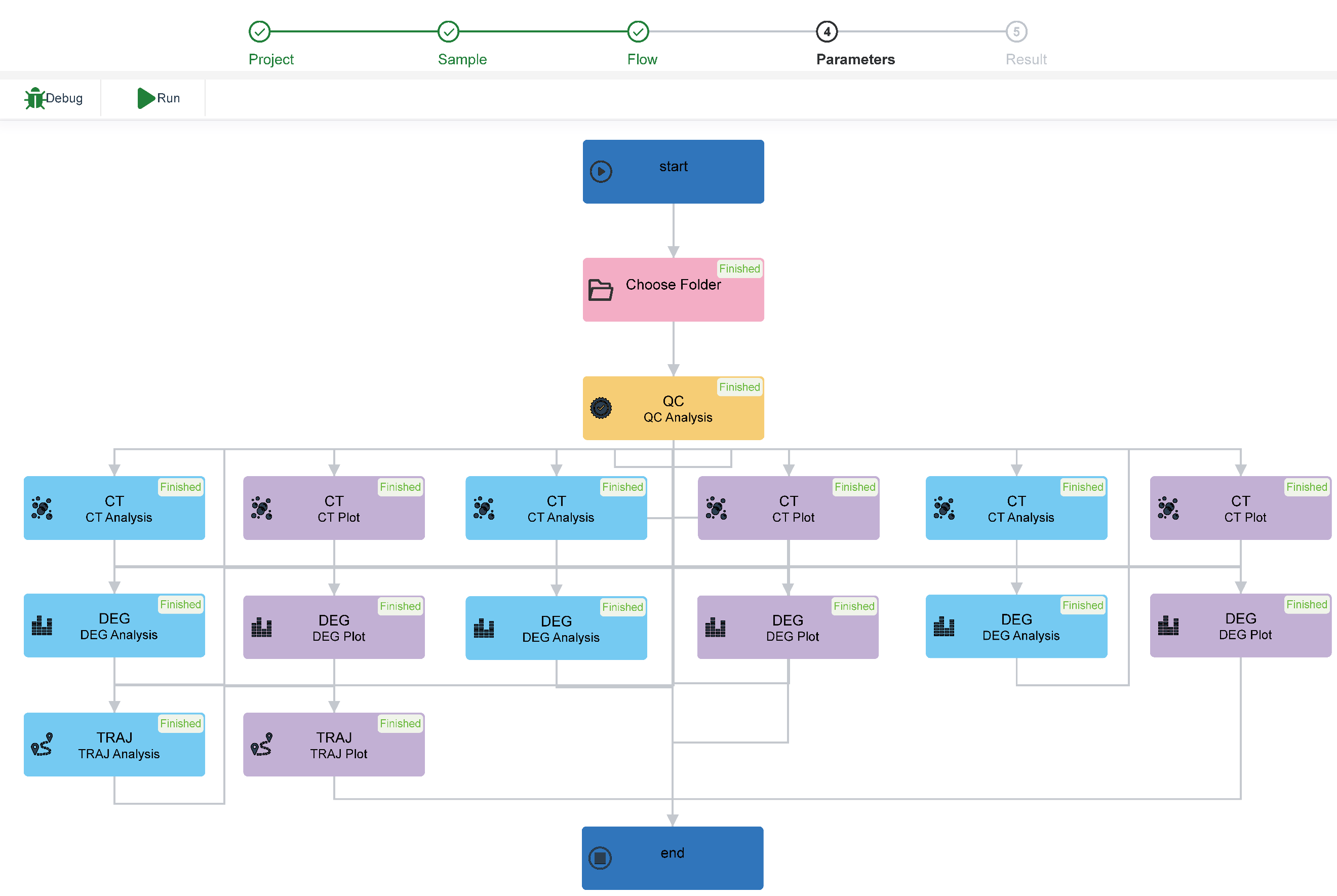


**Fig. S16 Pipeline of case study 3 in SRT-server.** We used QC, CT, DEG, and TRAJ module with its corresponding plot module. Note that we added three CT modules with different methods, which results in the output of DEG are different. TRAJ module is only followed by CT with BASS.


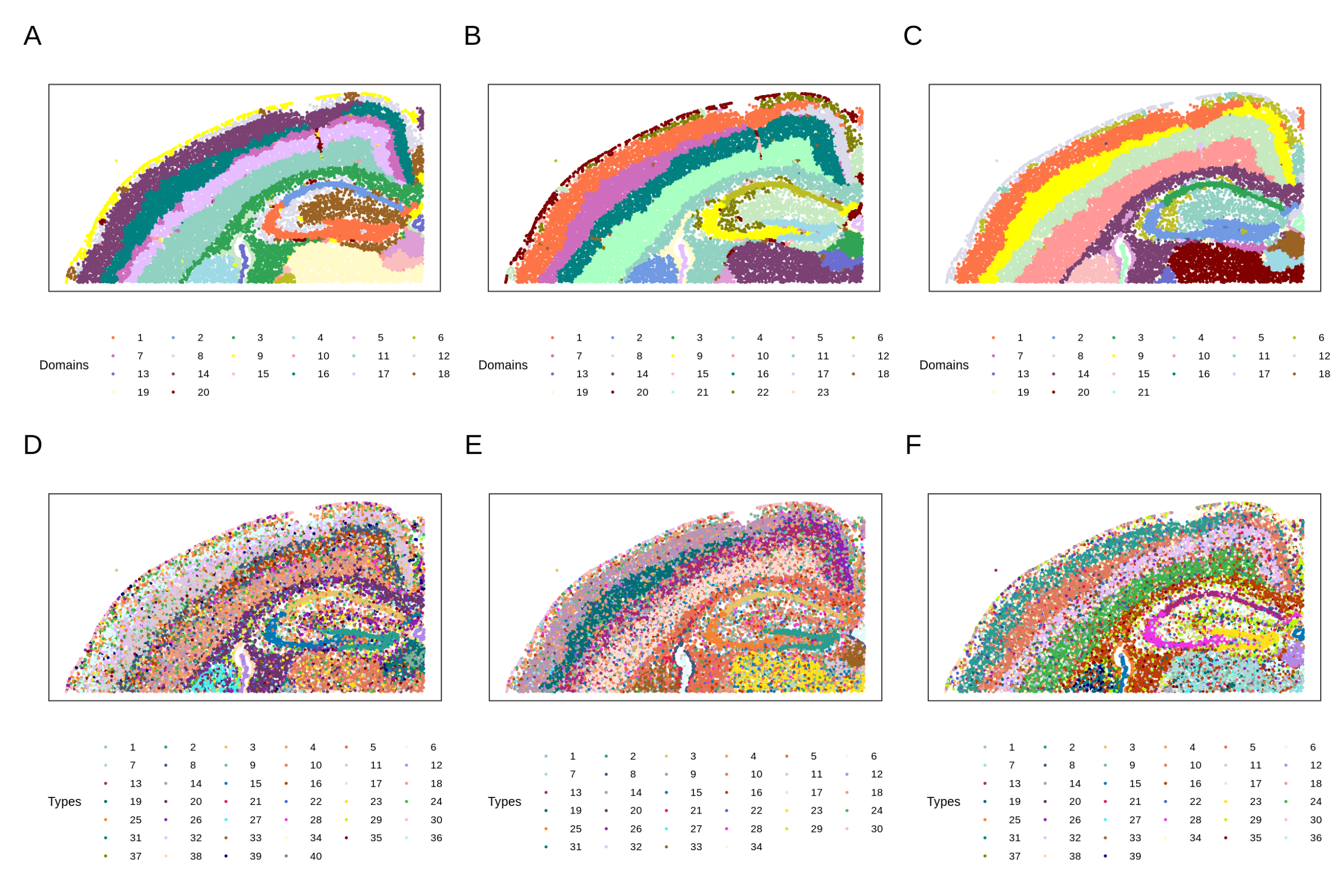


**Fig. S17 Summary for the CL_jo module with BASS.** A) The location plot for spatial domain with domain number = 20 and cell type number = 35. B) The location plot for spatial domain with domain number = 25 and cell type number = 35. C) The location plot for spatial domain with domain number = 25 and cell type number = 40. D) The location plot for cell type with domain number = 20 and cell type number = 35. E) The location plot for cell type with domain number = 25 and cell type number = 35. F) The location plot for cell type with domain number = 25 and cell type number = 40.


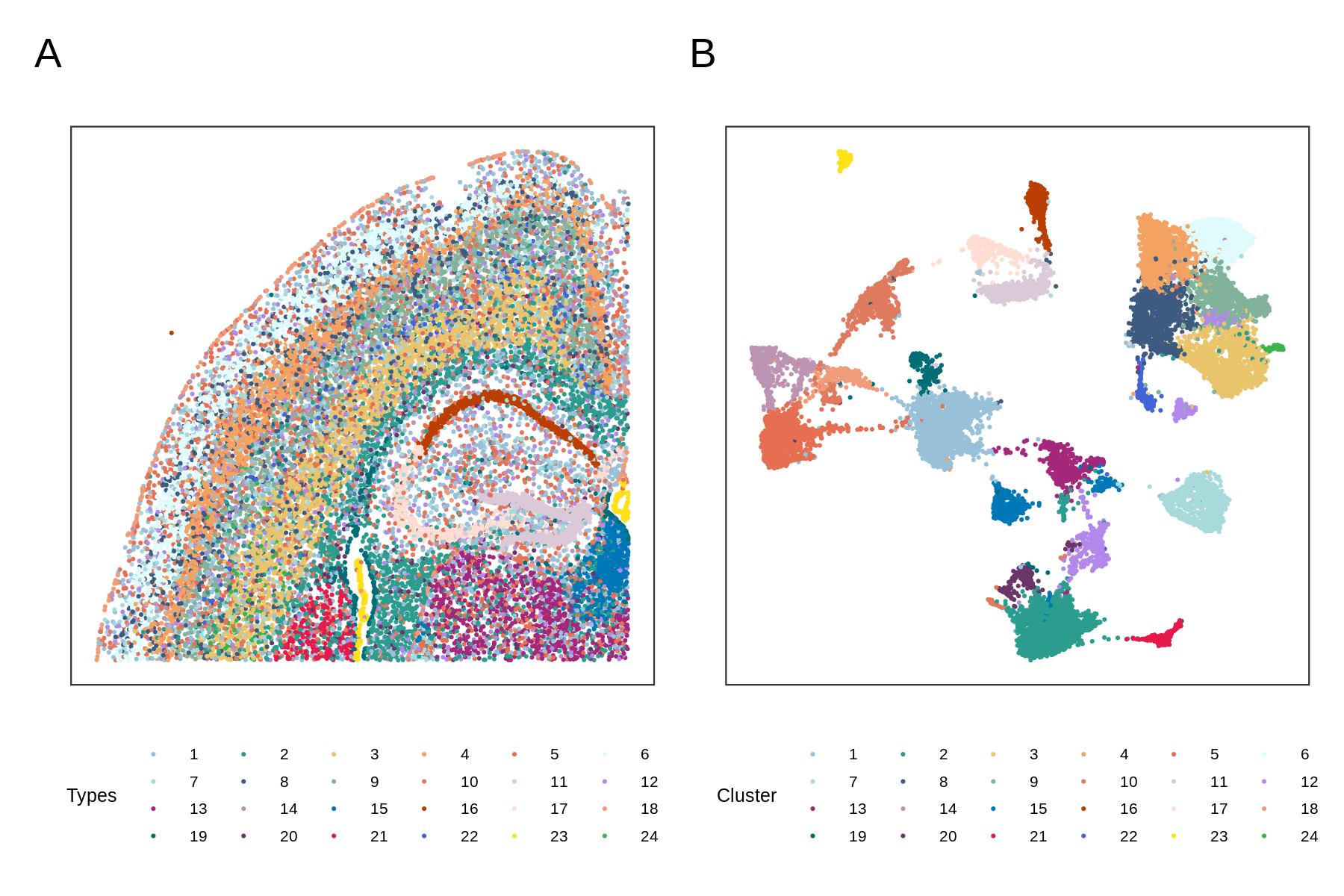


**Fig. S18 Summary for the clustering result from Seurat.** A) The location plot of cell type with resolution = 0.5. B) The UMAP plot with resolution = 0.5.


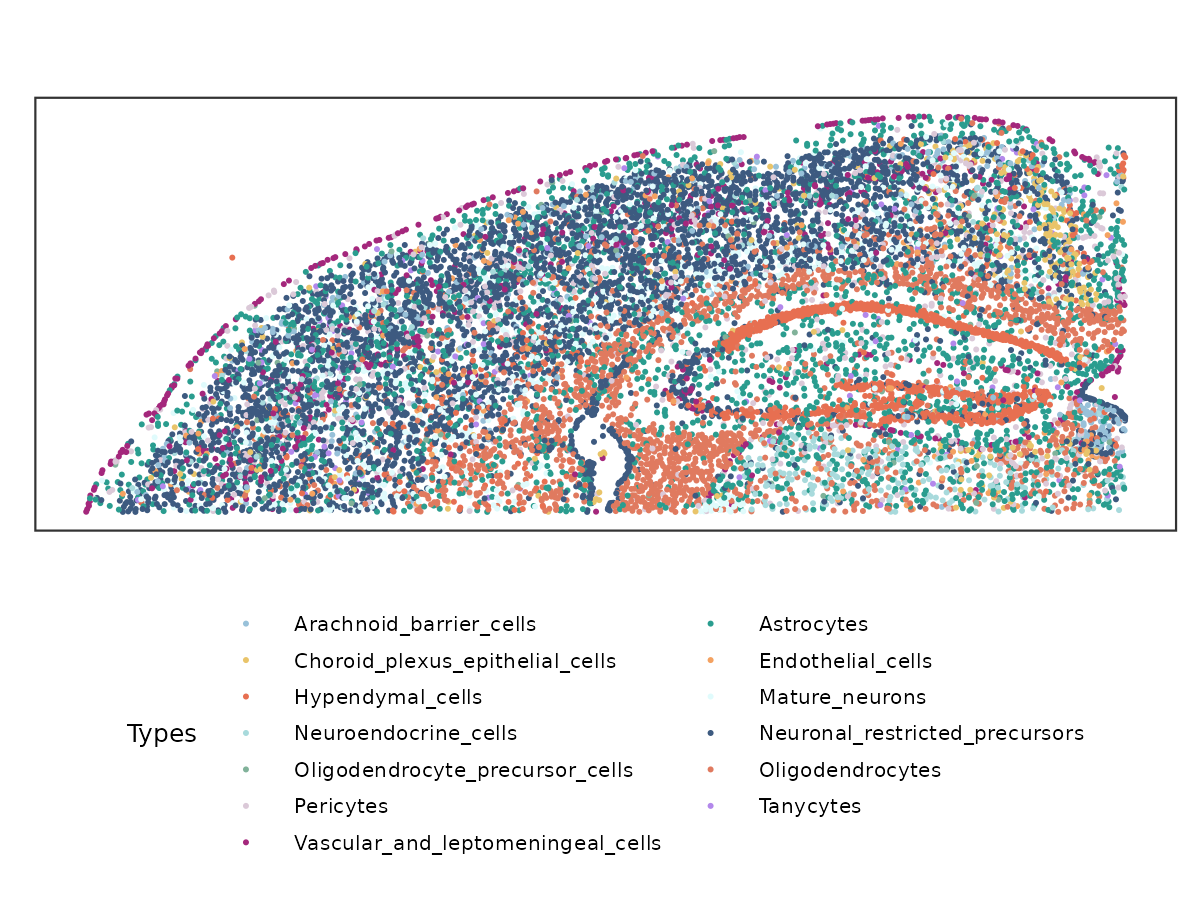


**Fig. S19 17 cell types annotated by Garnett in the CL_annot module.**


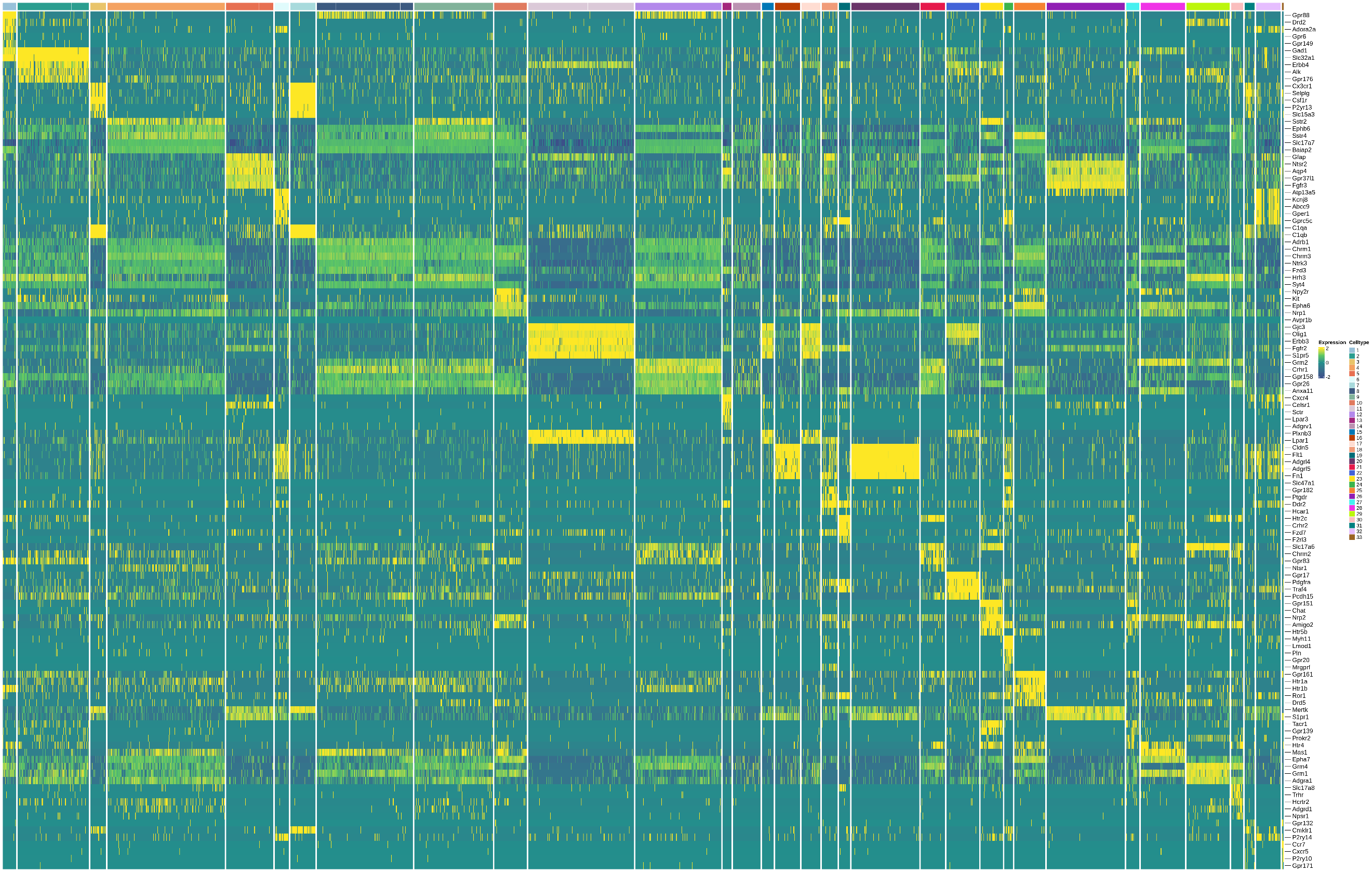


**Fig. S20 The Heatmap for BASS with top five DEGs.** We use BASS to cluster the cells into 33 domains. The heatmap shows the top five DEGs for each domain.

**
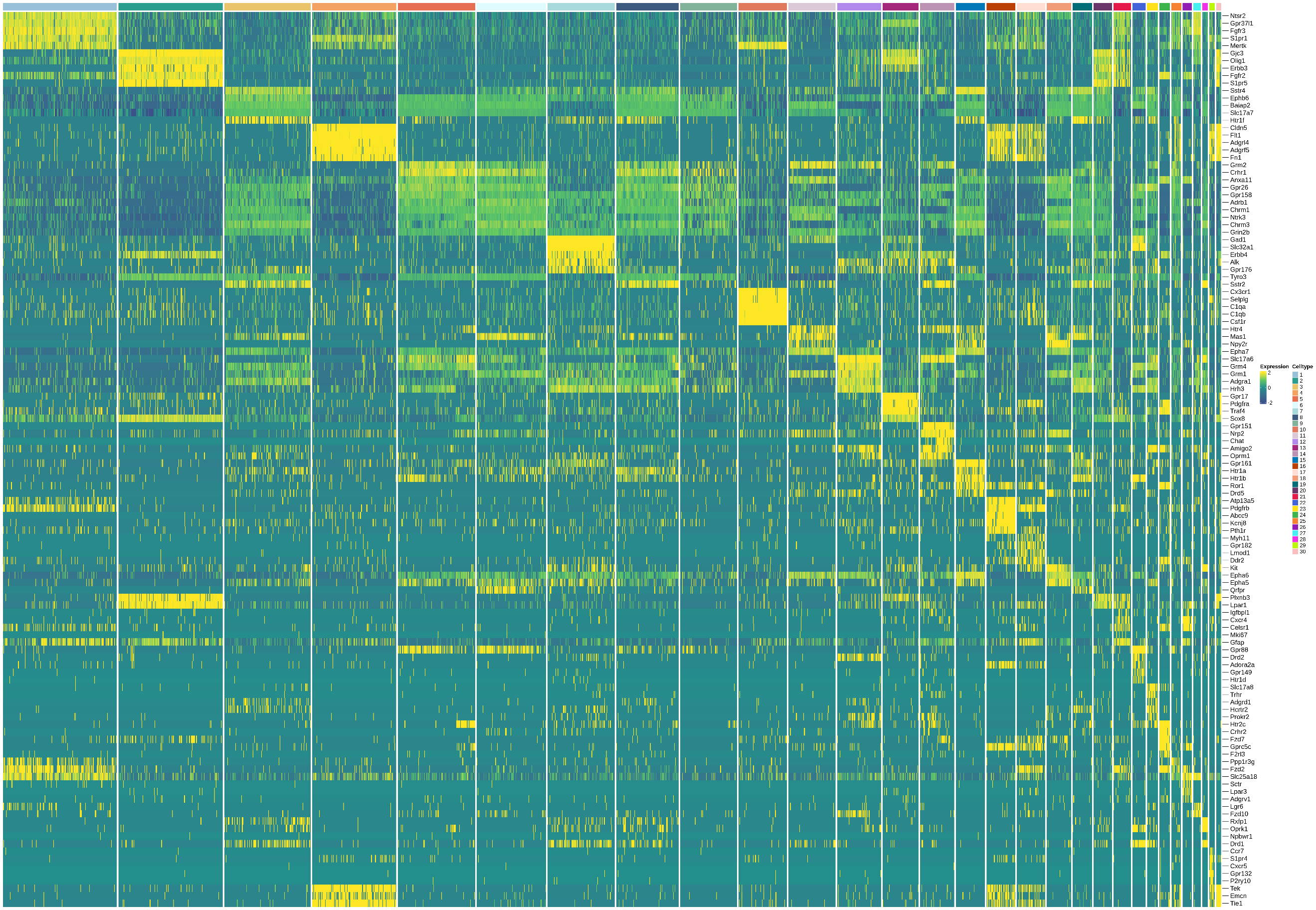
**

**Fig. S21 The Heatmap from Seurat (resolution = 1) with top five DEGs.** We use Seurat to cluster the cells into 30 cell types. The heatmap shows the top five DEGs for each domain.


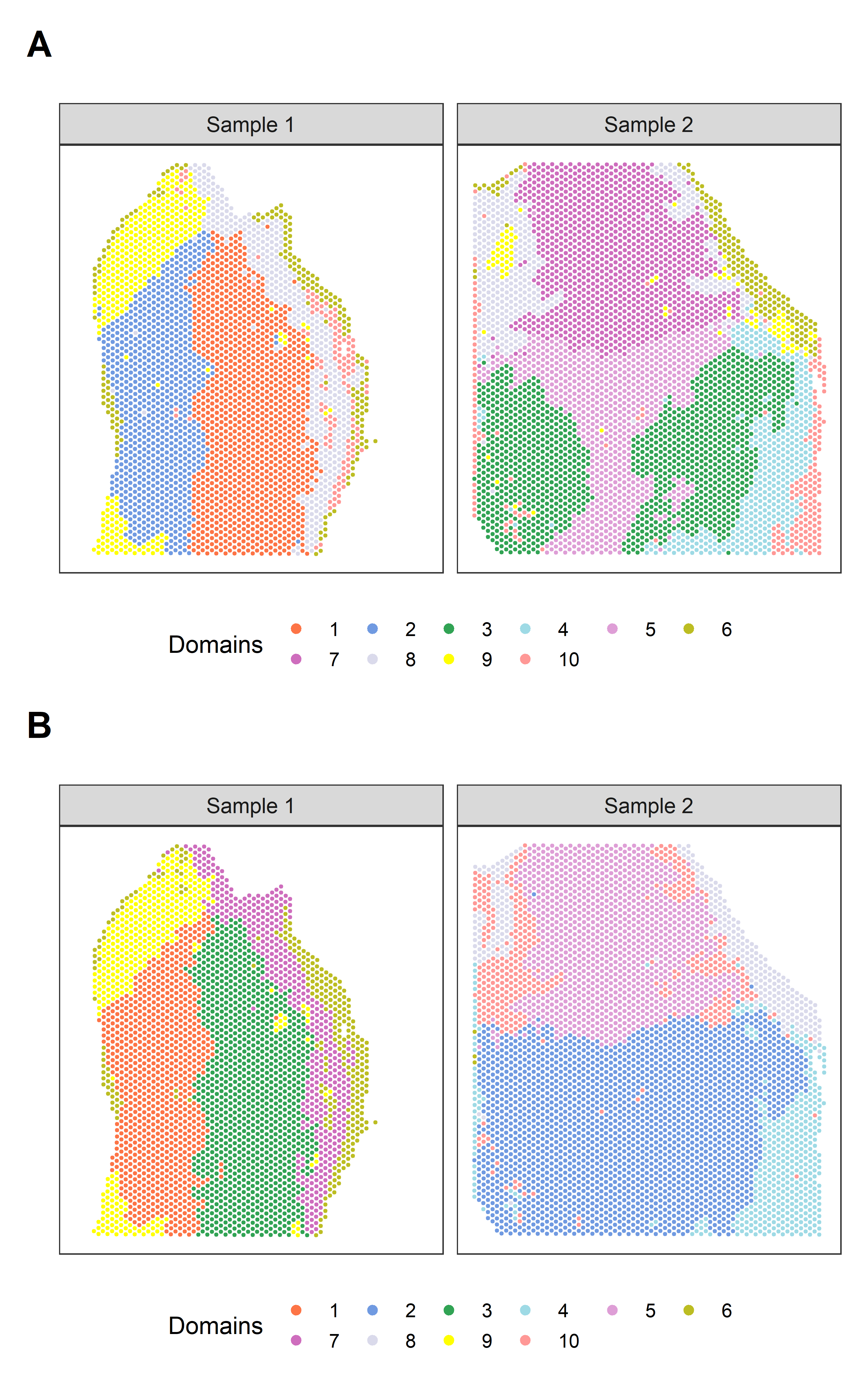


**Fig.** **S22 Location plots display the domain clustering results from BASS on different samples with or without the data integration step.** The number of spatial domains is set to be 10 and the number of cell types is set to be 20. A) BASS results after a data integration step with Harmony; B) BASS results without data integration.
